# Double-faced CX3CL1 enhances lymphangiogenesis-dependent metastasis in an aggressive subclone of oral squamous cell carcinoma

**DOI:** 10.1172/jci.insight.174618

**Published:** 2024-05-22

**Authors:** Htoo Shwe Eain, Hotaka Kawai, Masaaki Nakayama, May Wathone Oo, Toshiaki Ohara, Yoko Fukuhara, Kiyofumi Takabatake, Quisheng Shan, Yamin Soe, Kisho Ono, Keisuke Nakano, Nobuyoshi Mizukawa, Seiji Iida, Hitoshi Nagatsuka

**Affiliations:** 1 Department of Oral Pathology and Medicine,; 2 Department of Oral and Maxillofacial Reconstructive Surgery, and; 3 Department of Oral Microbiology, Graduate School of Medicine, Dentistry and Pharmaceutical Sciences, Okayama University, Okayama, Japan.; 4 Office of Innovative Medicine, Organization for Research Strategy and Development, Okayama University, Okayama, Japan.; 5 Department of Pathology and Experimental Medicine,; 6 Department of Oral Morphology, and; 7 Department of Oral and Maxillofacial Surgery, Graduate School of Medicine, Dentistry and Pharmaceutical Sciences, Okayama University, Okayama, Japan.

**Keywords:** Oncology, Chemokines, Head and neck cancer

## Abstract

Because cancer cells have a genetically unstable nature, they give rise to genetically different variant subclones inside a single tumor. Understanding cancer heterogeneity and subclone characteristics is crucial for developing more efficacious therapies. Oral squamous cell carcinoma (OSCC) is characterized by high heterogeneity and plasticity. On the other hand, CX3C motif ligand 1 (CX3CL1) is a double-faced chemokine with anti- and pro-tumor functions. Our study reported that CX3CL1 functioned differently in tumors with different cancer phenotypes, both in vivo and in vitro. Mouse OSCC 1 (MOC1) and MOC2 cells responded similarly to CX3CL1 in vitro. However, in vivo, CX3CL1 increased keratinization in indolent MOC1 cancer, while CX3CL1 promoted cervical lymphatic metastasis in aggressive MOC2 cancer. These outcomes were due to double-faced CX3CL1 effects on different immune microenvironments indolent and aggressive cancer created. Furthermore, we established that CX3CL1 promoted cancer metastasis via the lymphatic pathway by stimulating lymphangiogenesis and transendothelial migration of lymph-circulating tumor cells. CX3CL1 enrichment in lymphatic metastasis tissues was observed in aggressive murine and human cell lines. OSCC patient samples with CX3CL1 enrichment exhibited a strong correlation with lower overall survival rates and higher recurrence and distant metastasis rates. In conclusion, CX3CL1 is a pivotal factor that stimulates the metastasis of aggressive cancer subclones within the heterogeneous tumors to metastasize, and our study demonstrates the prognostic value of CX3CL1 enrichment in long-term monitoring in OSCC.

## Abstract 2

Syngeneic mouse models of oral cancer indicate that the double-faced chemokine influences lymphangiogenesis-dependent metastasis and has potential as a prognostic marker.

## Graphical abstract

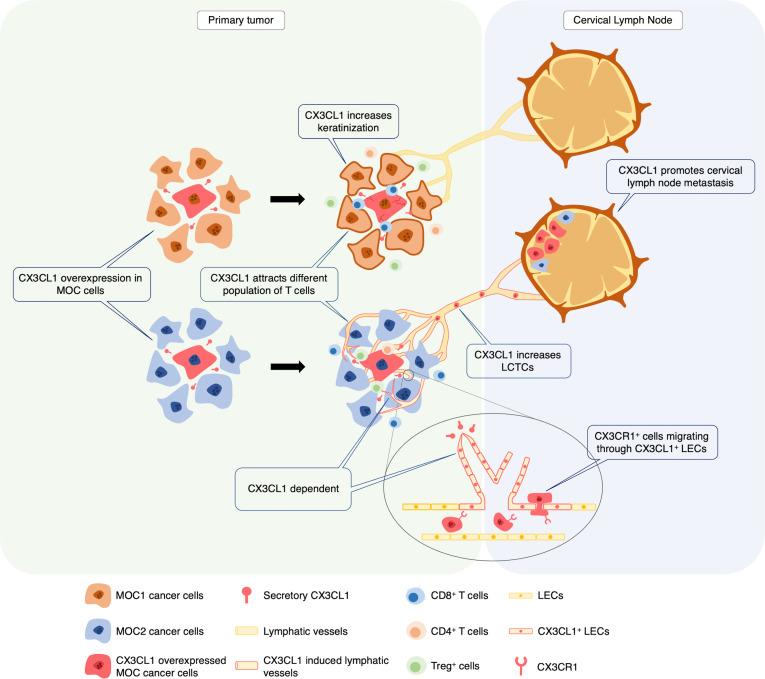

## Introduction

During cancer progression, the selection process is constantly occurring inside the tumor, giving rise to heterogenic populations of cancer subclones (1, 2). Cancer subclones have diverse genetic behaviors, creating phenotypically different tumor microenvironments (TMEs) that influence the course of disease progression and impact cancer treatments (3–5). Oral squamous cell carcinoma (OSCC) is a polyclonal cancer characterized by intratumoral heterogeneity and plasticity (6). Previous studies showed that the same cancer therapy in patients with OSCC can display variable responses. This may be due to various cancer subclone responses to cancer drug therapy.

Chemokines are small chemotactic cytokines with various effects on the TME. Some cytokines and chemokines have been successfully used as targets for drug therapy in treating cancer (7, 8). Despite successful treatment in most patients, some experienced little to no response and even adverse outcomes in the worst case scenario. These outcomes can vary because of the multifaceted roles of chemokines, including their tumor-inhibiting and tumor-promoting properties. The functions of chemokines range from altering cancer characteristics to regulating immune cell trafficking into the TME (9). In addition, some chemokines change the tumor niche architecture, such as vascular remodeling (10). Therefore, it is essential to evaluate the effects of chemokines on different cancer subclones in vitro and in vivo. Our study revealed that CX3C motif chemokine ligand 1 (CX3CL1) had a higher expression in the metastatic phenotypic cancer mouse OSCC 2 (MOC2) than in MOC1, which had a localized tumor phenotype.

CX3CL1 is the sole member of the CX3C motif chemokine family member with a high affinity for the receptor CX3CR1 (11). CX3CL1 increases the infiltration of lymphocytes, including cytotoxic T cells, NK cells, and dendritic cells (DCs), known for their antitumor effects (12). DCs are known to express CX3CL1, which promotes the infiltration of CD4^+^ T cells and CD8^+^ T cells into the tumors, improving prognosis (13). Conversely, the CX3CL1/CX3CR1 axis has been shown to promote cancer cell proliferation, migration, and invasion, along with recruiting pro-tumor immune cells (14, 15). CX3CL1 also promotes tumor vascularization directly or indirectly via tumor-associated macrophages (16–18). CX3CL1 is unique because of its N-terminal domain containing a CX3C motif and a long mucin body, with each chemokine domain playing distinct roles, such as signaling and adhesion (19). Given its multifaceted roles in the TME and complex molecular structures, the precise role of CX3CL1 in OSCC remains unclear. For this reason, in addition to using CX3CL1 overexpression models, we created the signal peptide–mutant form and chemokine domain–mutant form of CX3CL1 to further analyze the detailed functions of CX3CL1 on OSCC.

MOC1 and MOC2 are the syngeneic murine OSCC cell lines derived from the same background, but with different characteristics, suitable for injection into immunocompetent mice (20). MOC1 forms localized tumors with well-differentiated features and an immune-inflamed phenotype, whereas MOC2 is a poorly differentiated, aggressive metastatic cancer with an immune-excluded phenotype (20, 21).

Using orthotopic syngeneic cancer cell lines in naturally occurring tumor immune microenvironments, we could observe the full capacity of chemokine CX3CL1 in cancer cells, immune cells, and tumor-supporting structures in the TME.

In this study, we reported the different effects of CX3CL1 on MOC1 and MOC2, representing progressive OSCC with a diverse nature. We also elucidated the mechanism by which CX3CL1 stimulated aggressive subclones to promote metastasis via the lymphatic pathway.

## Results

### Comprehensive analysis of syngeneic OSCC tumors reveals the chemokine-related genes, and CX3CL1 is the chemokine-associated upregulated gene in aggressive MOC tumors.

**Figure 1:**
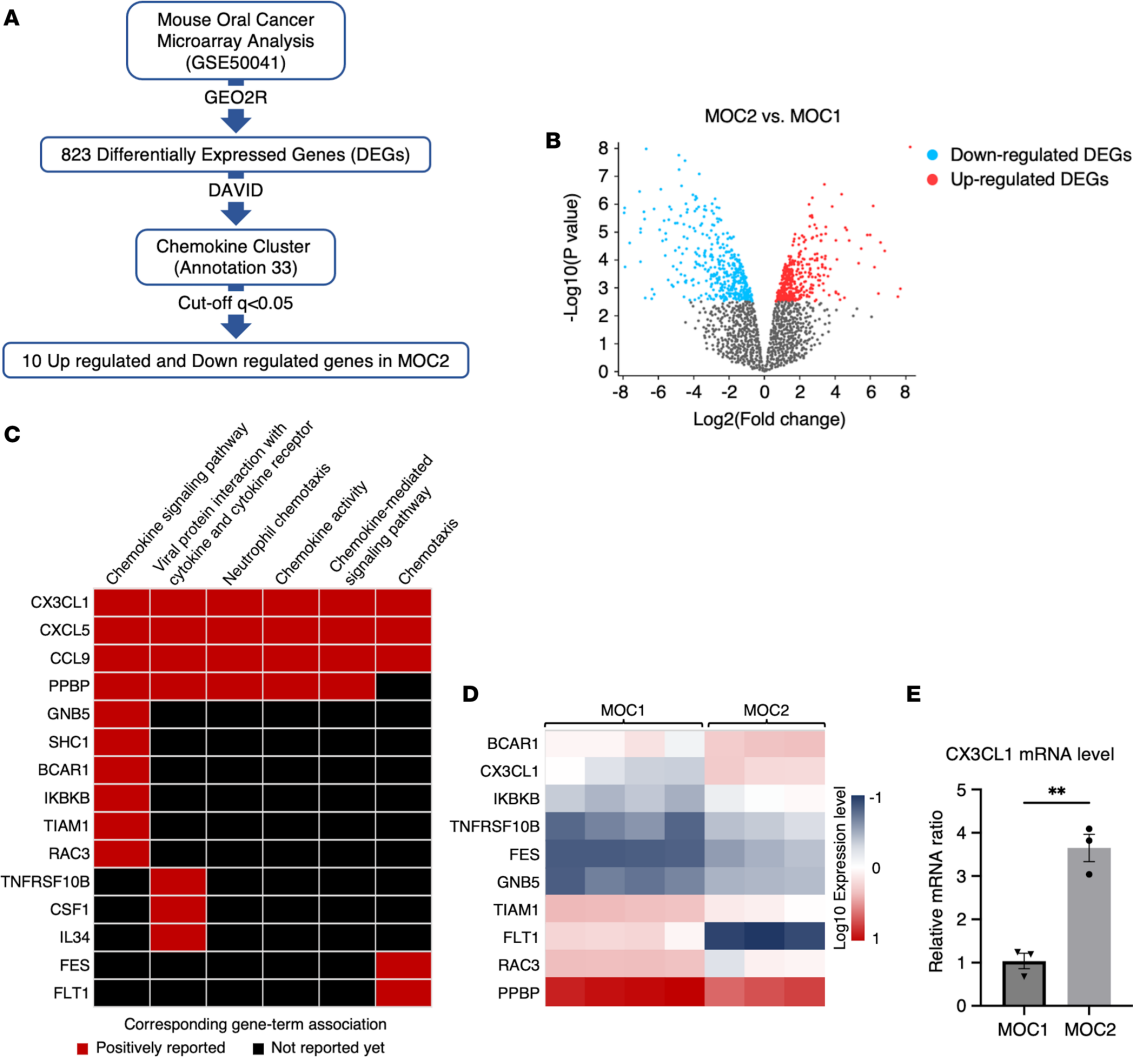
*Cx3cl1* is the upregulated gene in aggressive syngeneic mouse OSCC tumors. MOC1 and MOC2 data analysis was done, and the chemokine-associated genes upregulated in MOC2 were selected. (**A**) Flowchart of chemokine-related gene analysis in MOC cell lines. We selected the differentially expressed genes (DEGs) of MOC using GEO2R. Then, we used the Database for Annotation, Visualization and Integrated Discovery (DAVID) software to choose the genes related to chemokine and chemotaxis functions with a cutoff line at *q* < 0.05 and identified chemokine-related genes in MOC2. (**B**) Volcano plot analysis showing upregulated and downregulated DEGs in MOC2 versus MOC1 of GEO data (GSE50041). The blue dots represent the downregulated DEGs, and the red dots represent the upregulated DEGs. Padj < 0.05. (**C**) Heatmap summary of genes from chemokine-related categories from DAVID functional enrichment analysis. (**D**) Heatmap summary of the expression level of chemokine-related genes with *q* < 0.05 in MOC2 versus MOC1. (**E**) Relative mRNA expression of *Cx3cl1* in MOC2 versus MOC1 was determined by quantitative reverse-transcriptase PCR (RT-qPCR). *Cx3cl1* expression was analyzed by the 2^–ΔΔCt^ method. *Cx3cl1* expression level in MOC1 was set as 1. (*n* = 3.) All data are presented as mean ± SEM. Statistical analysis was done using Student’s *t* test. **P < 0.01.

To identify the chemokine-related genes in MOC cell lines, we performed a functional analysis of the gene expression data sets of MOC cell lines that were publicly available in the National Center for Biotechnology (NCBI) Gene Expression Omnibus (GEO) database (accession no. GSE50041) (Figure 1A). To identify DEGs from MOC cell lines, we used GEO2R analysis software with the cutoff line of adjacent *P* value less than 0.05 and identified 823 DEGs (Figure 1B). Chemokines are chemotactic cytokines that influence cancer cells directly or via signaling (22). We further performed functional analysis of DEGs using the DAVID functional annotation tool to select the chemokine- and chemotaxis-related genes, obtaining 15 genes associated with chemokine functions. Notably, *Cx3cl1*, *Cxcl5*, and *Ccl9* were positively correlated with all chemokine- and chemotaxis-related functions (Figure 1C).

To refine our selection, we established a cutoff line with a *q* value less than 0.05. Among the chemokine-related genes associated with all categories, *Cx3cl1* emerged as a significantly upregulated gene in aggressive MOC2 cancer (Figure 1D).

To verify the expression level of *Cx3cl1* in MOC1 and MOC2 cell lines, we assessed the relative mRNA expression of *Cx3cl1*. Our finding revealed that the mRNA expression level of *Cx3cl1* was higher in MOC2 cells than in MOC1 cells (Figure 1E).

To analyze the characteristics of MOC cells, we examined cell shapes and cell-to-cell contact. Desmosomal attachments play a vital role in adhesion between epithelium cells (23), with their loss commonly observed in invasive cancer cells (24). We observed desmosomal structures between MOC1 cells, whereas MOC2 cells displayed minimal desmosomal structures (Supplemental Figure 1A; supplemental material available online with this article; https://doi.org/10.1172/jci.insight.174618DS1). Based on these results, we concluded that MOC2 cells may have more invasive properties than MOC1 cells.

To verify the in vivo characteristics and nature of MOC tumors, we established the orthotopic tumor models by inoculating MOC1 and MOC2 tumors into the buccal mucosa of C57BL/6J female mice. MOC1 tumors exhibited well-differentiated characteristics, whereas MOC2 tumors displayed poorly differentiated characteristics. Naturally occurring metastases to cervical lymph nodes (LNs) and lungs were observed in MOC2 tumors, while there were no lymphatic metastases in MOC1 tumors, suggesting that MOC1 is a nonmetastatic, indolent cancer type and that MOC2 is an aggressive, metastasizing cancer type (Supplemental Figure 1B). To check the tissue expression of CX3CL1 in the TME, we immunostained and counted CX3CL1^+^ cells in the primary tumor tissues of MOC1 and MOC2. The number of CX3CL1^+^ cells was significantly higher in the MOC2 TME, consistent with microarray and reverse transcription quantitative PCR analysis (Supplemental Figure 1, C and D).

These results indicate that *Cx3cl1* is the chemokine and chemotaxis-related gene upregulated in metastatic cancer.

### CX3CL1 increases cell migration, inhibits the cell proliferation rate of both MOC cells in vitro, and can recruit CX3CR1^+^ cells into the TME.

**Figure 2:**
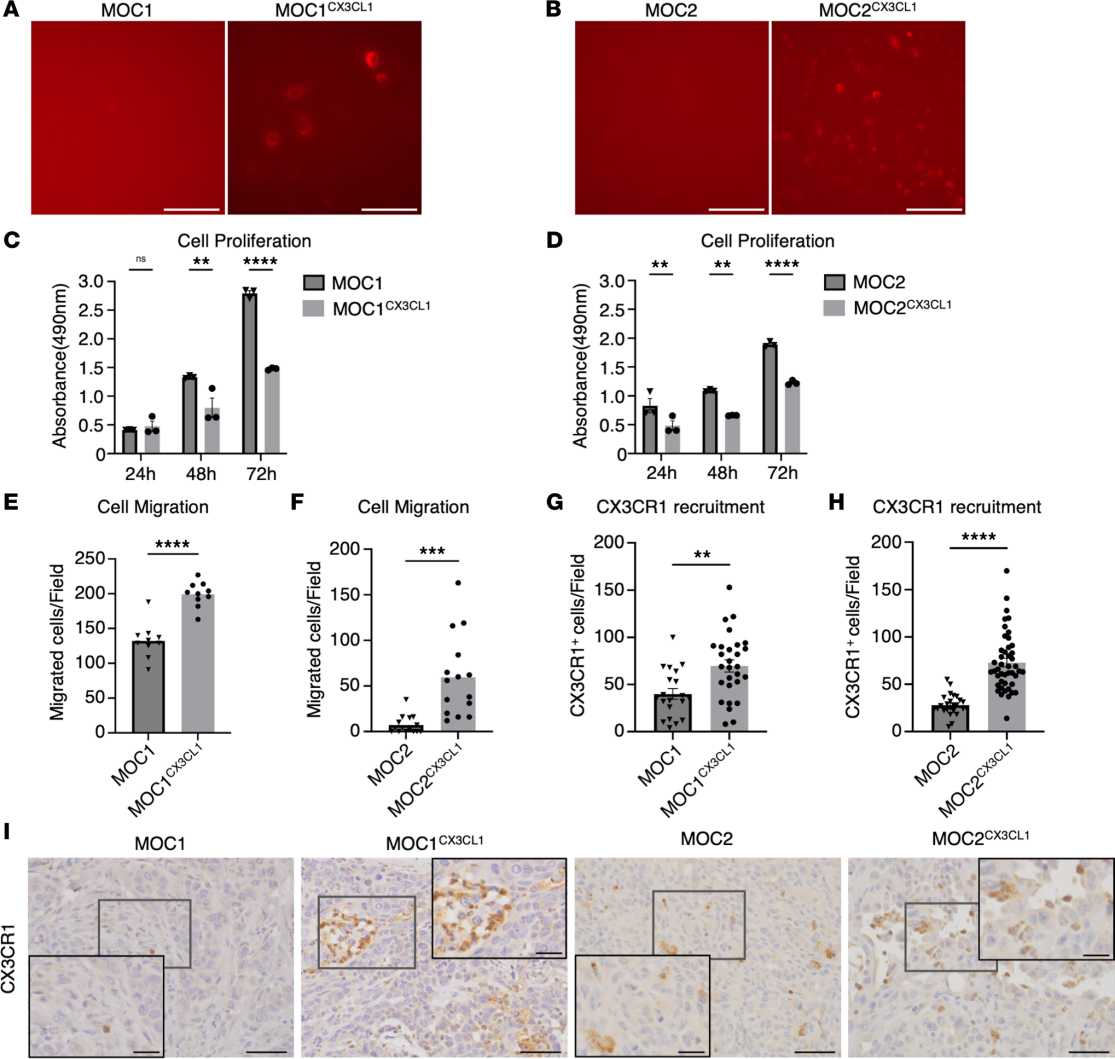
CX3CL1 increases cell migration, inhibits the cell proliferation rate of both MOC OSCC cells in vitro, and can recruit CX3CR1^+^ cells into the TME. CX3CL1 was overexpressed in MOC1 and MOC2, and FLAG-DsRed expression was tagged to the overexpressed cells. CX3CL1 overexpression was confirmed using FLAG expression. Representative images of FLAG expression in (**A**) MOC1 and MOC1^CX3CL1^ and (**B**) MOC2 and MOC2^CX3CL1^ cells. Scale bar: 100 μm. MTS assay was used to measure the cell proliferation rate between MOC and MOC^CX3CL1^ cells. Cell proliferation rate between (**C**) MOC1 and MOC1^CX3CL1^ and (**D**) MOC2 and MOC2^CX3CL1^ at 24 hours, 48 hours, and 72 hours. (*n* = 3.) We used the Transwell migration assay to assess the migration ability of the MOC cells after CX3CL1 overexpression. The number of migrating cells per field in (**E**) MOC1 and MOC1^CX3CL1^ and (**F**) MOC2 and MOC2^CX3CL1^. (*n* = 3.) The CX3CL1/CX3CR1 axis has an autocrine function, and we verified the recruitment of CX3CR1^+^ cells into the TME of MOC tumors. The number of CX3CR1^+^ cells per field in (**G**) MOC1 and MOC1^CX3CL1^ tumors and (**H**) MOC2 and MOC2^CX3CL1^ tumors. (**I**) Representative images showing recruited CX3CR1^+^ cells into MOC and MOC^CX3CL1^ tumors. Scale bar: 50 μm, scale bar (insets): 20 μm. MOC1, MOC1 only; MOC1^CX3CL1^, MOC1 with CX3CL1 overexpression; MOC2, MOC2 only; MOC2^CX3CL1^, MOC2 with CX3CL1 overexpression. MOC1: *n* = 4, MOC1^CX3CL1^: *n* = 6, MOC2: *n* = 5, MOC2^CX3CL1^: *n* = 9. All data are presented as mean ± SEM. Statistical analysis was done using Student’s *t* test to compare 2 groups and 1-way ANOVA followed by Tukey’s multiple-comparison post hoc test for comparison of 3 groups or more. *P < 0.05, **P < 0.01, ***P < 0.001, ****P < 0.0001.

To investigate the functional roles of CX3CL1 on different OSCC phenotypes, we created the CX3CL1 overexpression models of MOC cell lines using plasmid transfection. The overexpressing cell lines were termed MOC1^CX3CL1^ and MOC2^CX3CL1^. The overexpression plasmids were tagged with the DYKDDDDK (FLAG) epitope and the DsRed fluorescent reporter protein (FLAG-DsRed). Transfected cells were selected using the puromycin selection method. To confirm the success of transfection, FLAG-DsRed expression was examined under a confocal microscope, and overexpression was confirmed (Figure 2, A and B).

To identify the influence of CX3CL1 on the characteristics of MOC cancer cells, we performed MTS and Transwell migration assays to evaluate cell proliferation and migration efficacy, respectively. We found that MOC1^CX3CL1^ and MOC2^CX3CL1^ cells had significantly lower cell proliferation rates than MOC1 and MOC2 cells (Figure 2, C and D). However, the migratory abilities of MOC1^CX3CL1^ and MOC2^CX3CL1^ were significantly enhanced after CX3CL1 overexpression (Figure 2, E and F).

These data suggest that CX3CL1 inhibits the proliferation and promotes the migration of MOC cells in vitro.

To further investigate the in vivo effects of CX3CL1 on the MOC tumors, we created orthotopic murine models of CX3CL1-overexpressed cells in the same manner as their corresponding MOC tumors. The primary tumor tissues were then immunostained and counted to confirm CX3CL1 overexpression in vivo. The overexpression models, MOC1^CX3CL1^ and MOC2^CX3CL1^, had a significantly higher number of CX3CL1^+^ cells than the nontransfected models (Supplemental Figure 2, A–C). These results verified the overexpression of CX3CL1 in the TME of MOC1 and MOC2 tumors.

CX3CR1 is a high-affinity chemokine receptor of CX3CL1 that is expressed in immune cells such as T cells, NK cells, and macrophages, as well as in some cancer cells (25). To investigate whether CX3CL1 overexpression in cancer cells can attract the CX3CR1^+^ cells into the TME, we stained and counted the CX3CR1^+^ cells in the TME of MOC1 and MOC2 tumors. CX3CR1^+^ cells were increased in MOC^CX3CL1^ tumors compared with their respective MOC tumors (Figure 2, G–I).

These results indicate that CX3CR1^+^ cells are recruited into the TME of MOC tumors after CX3CL1 overexpression.

### The influences of CX3CL1 on nonmetastatic and metastatic tumors.

**Figure 3:**
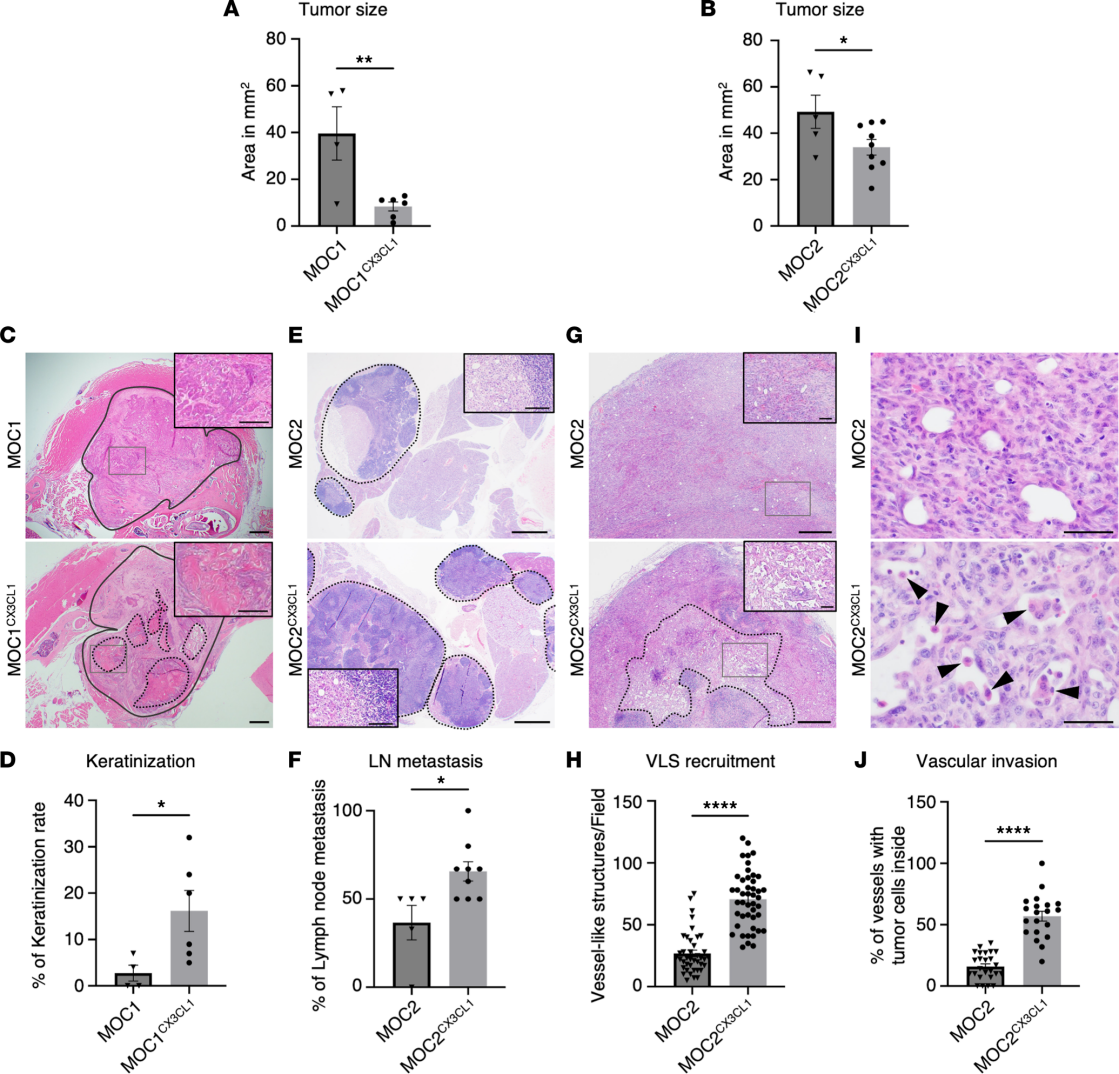
The influences of CX3CL1 on nonmetastatic and metastatic tumors. Tumor sizes of (**A**) MOC1 and MOC1^CX3CL1^ tumors and (**B**) MOC2 and MOC2^CX3CL1^ tumors measured by area in square millimeters. (**C**) Representative images showing histological changes in keratinization in the primary tumors of MOC1 and MOC1^CX3CL1^ tumors. The solid line encloses the tumor area. Dotted lines represent the keratinized areas in MOC1^CX3CL1^ tumors. Scale bar: 500 μm. The magnified images of the tumor center are shown in the insets. Scale bar (insets): 250 μm. (**D**) The percentage of keratinization area in MOC1 and MOC1^CX3CL1^ tumors. (**E**) Representative images of MOC2 and MOC2^CX3CL1^ LNs. Dotted lines represent LNs. Scale bar: 1 mm. The magnified images of the LN metastasis area are shown in the insets. Scale bar (insets): 100 μm. (**F**) The metastasis rate of MOC2 and MOC2^CX3CL1^ LNs. (**G**) Representative images showing histological changes in vasculature in the primary tumors of MOC2 and MOC2^CX3CL1^ tumors. Dotted line represents the densely vascular area. Scale bar: 500 μm. The magnified images of the tumor center are shown in the insets. Scale bar (insets): 100 μm. (**H**) The number of VLSs per field in MOC2 and MOC2^CX3CL1^ tumors. (**I**) Representative image of VLSs containing tumor cells in MOC2 and MOC2^CX3CL1^ tumors. Arrows indicate tumor cells. Scale bar: 50 μm. (**J**) The percentage of VLSs containing tumor cells in MOC2 and MOC2^CX3CL1^ tumors. MOC1: *n* = 4, MOC1^CX3CL1^: *n* = 6, MOC2: *n* = 5, MOC2^CX3CL1^: *n* = 9. All data are presented as mean ± SEM. Statistical analysis was done using Student’s *t* test. *P < 0.05, **P < 0.01, ****P < 0.0001.

To further investigate the in vivo effects of CX3CL1 on cancer, we analyzed various areas that could predict cancer outcomes, including tumor size, keratinization, and metastasis (Table 1). First, we examined the tumor size, measured as the area in square millimeters. The tumor sizes of MOC1^CX3CL1^ and MOC2^CX3CL1^ were significantly smaller than those of the corresponding MOC1 and MOC2 tumors (Figure 3, A and B). These in vivo results correlated with the in vitro results, in which the cell proliferative abilities of MOC1^CX3CL1^ and MOC2^CX3CL1^ were significantly reduced. These results show that CX3CL1 reduces the cell proliferative ability of MOC2 cells both in vitro and in vivo.

On closer examination of the characteristics of MOC1^CX3CL1^ and MOC2^CX3CL1^ primary tumors, we discovered that the histopathological features of MOC1^CX3CL1^ and MOC2^CX3CL1^ tumors were completely different from MOC1 and MOC2 tumors. Increased levels of CX3CL1 in the MOC1 tumors showed a drastic change in cell differentiation compared with MOC1 tumors. Increased keratinization in the OSCC indicates better clinical results in patients with OSCC (26). The primary tumor of MOC1^CX3CL1^ showed an increase in keratinized island areas (Figure 3, C and D). However, CX3CL1 did not influence the metastatic ability of the indolent, nonmetastatic MOC1 cancer cells (data not shown). In contrast, MOC2^CX3CL1^ tumors showed a significant increase in LN metastasis compared with MOC2 tumors (Figure 3, E and F).

These data suggest that the chemokine CX3CL1 promotes the metastatic ability of aggressive cancer and cell differentiation of indolent cancer. However, CX3CL1 does not influence indolent cancer metastasis.

To identify the cause of increased cervical LN metastasis in MOC2^CX3CL1^ tumors, we closely analyzed the histological changes between MOC2 and MOC2^CX3CL1^ tumors’ tissues. In addition to the increasing cervical LN metastasis, tumor angiogenesis and lymphangiogenesis are prominent features of a worsening prognosis (27). MOC2^CX3CL1^ tumors showed increased formation of vessel-like structures (VLSs) in the tumor centers compared with MOC2 tumors (Figure 3, G and H). Vascular invasion and lymphovascular invasion are associated with higher recurrence rates and shorter survival time in patients with OSCC (28). We discovered that MOC2^CX3CL1^ tumors showed notably increased tumor cell invasion inside the VLSs (Figure 3, I and J). These tumor cells inside the VLSs indicated a higher metastatic potential of the primary tumors.

These results suggest that the chemokine CX3CL1 promotes cancer metastasis by creating the VLSs inside tumors and stimulating cancer cell invasion into the VLSs.

To investigate the discrepancy between the prognostic outcomes of MOC1^CX3CL1^ and MOC2^CX3CL1^, we examined changes in the immune landscape of CX3CL1-overexpressed tumors. CD4^+^ Th cells and FOXP3^+^ Treg cells are known to have immunosuppressive functions, whereas cytotoxic CD8^+^ T cells generally express antitumor ability (29, 30). We analyzed the T cell population recruited to the TME of MOC1^CX3CL1^ and MOC2^CX3CL1^ and found that CX3CL1 overexpression in MOC1 tumors decreased FOXP3^+^ Treg cells and increased CD8^+^ T cells’ infiltration. However, there was no significant change in CD4^+^ T cell infiltration (Supplemental Figure 3, A–C). MOC2^CX3CL1^ showed an increase in immunosuppressive CD4^+^ T cells and FOXP3^+^ Treg cells, with a considerable reduction in CD8^+^ T cells, compared with MOC2 (Supplemental Figure 3, D–F).

These data show that CX3CL1 promotes the recruitment of T cells into the TME. However, the recruited immune cell population may differ depending on the immunophenotypes of the TME created by indolent and aggressive cancer cells, resulting in a discrepancy in prognostic outcomes between the overexpression models.

### CX3CL1-overexpressed, aggressive OSCC cells metastasize to the cervical LN in a lymphangiogenesis-dependent manner.

**Figure 4:**
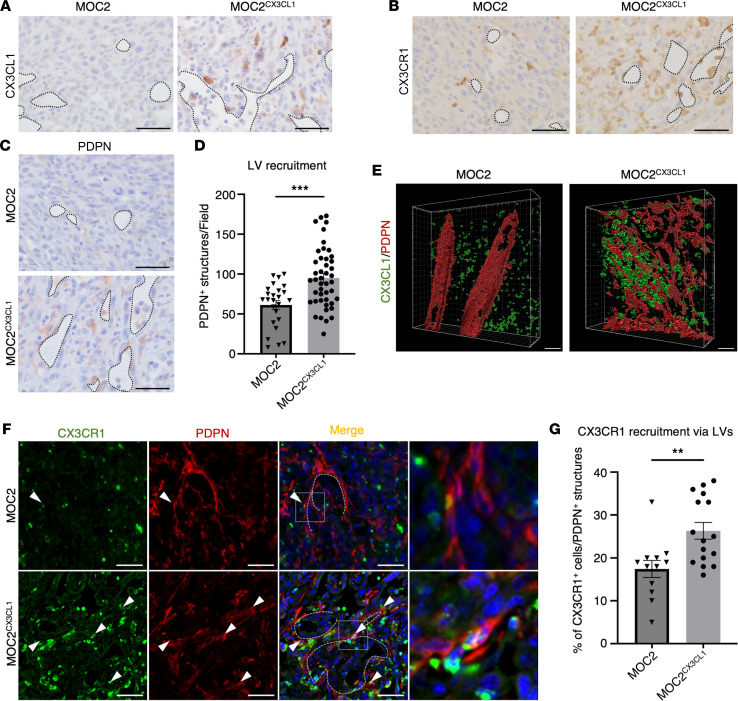
CX3CL1-overexpressed, aggressive OSCC cells metastasize to the cervical LNs via the CX3CL1/CX3CR1 axis in a lymphangiogenesis-dependent manner. Representative images showing (**A**) CX3CL1^+^ structures, (**B**) CX3CR1^+^ cells, and (**C**) PDPN^+^ structures accumulating around the VLSs in MOC2 and MOC2^CX3CL1^ tumors. Dotted lines represent VLSs. Scale bar: 50 μm. (**D**) The number of PDPN^+^ structures around the VLSs per field in MOC2 and MOC2^CX3CL1^ tumors. (**E**) Representative images showing double-fluorescence 3D staining of PDPN (red) and CX3CL1 (green). We verified the change in PDPN^+^ LV structures and an increased level of CX3CL1 in the TME of MOC2 and MOC2^CX3CL1^ tumors. Scale bar: 50 μm. (**F**) Representative images of double-fluorescence staining of CX3CR1 (green) and PDPN (red) in MOC2 and MOC2^CX3CL1^ tumors. We found the increased infiltration of CX3CR1^+^ cells into the PDPN^+^ LVs. Dotted lines represent PDPN^+^ LVs, and arrows represent merged cells. Scale bar: 50 μm. For far-right insets, the scale bar will be 10 µm. (**G**) The percentage of CX3CR1^+^ cells merging with PDPN^+^ structures in MOC2 and MOC2^CX3CL1^ tumors. MOC2: *n* = 5, MOC2^CX3CL1^: *n* = 9. All data are presented as mean ± SEM. Statistical analysis was done using Student’s *t* test. **P < 0.01, ***P < 0.001.

To identify the relationship between increasing VLSs and CX3CL1, we analyzed the histopathological locations of increasing CX3CL1^+^ and CX3CR1^+^ structures in MOC2^CX3CL1^ tumor tissues. We discovered that CX3CL1^+^ structures mainly accumulated and were positive around the VLSs in MOC2^CX3CL1^ tumors compared with MOC2 tumors (Figure 4A). Similarly, CX3CR1^+^ structures also accumulated around VLSs (Figure 4B).

These findings indicate that CX3CL1 and CX3CR1 may be positively correlated with increasing VLSs in MOC2^CX3CL1^ tumors.

Blood vessel and lymphatic vessel (LV) invasions in the TME are predictive indicators of OSCC prognosis (31, 32). To investigate the identity of the increased VLSs in MOC2^CX3CL1^ tumors, we immunostained the tumor tissue using the blood vessel marker, CD34, and the LV marker, podoplanin (PDPN) (33, 34). MOC2^CX3CL1^ primary tumors showed a significant increase in PDPN^+^ LV structures compared with MOC2 tumors (Figure 4, C and D). In contrast, we discovered that CD34^+^ blood vessel structures were fewer in MOC2^CX3CL1^ tumors than in MOC2 tumors, indicating that angiogenesis had little to no association with increasing VLSs in MOC2^CX3CL1^ tumors (Supplemental Figure 4, A and B).

These results suggest that the increasing VLSs inside MOC2^CX3CL1^ tumors are PDPN^+^ LVs.

To assess the structural association between CX3CL1^+^, CX3CR1^+^, and PDPN^+^ cells, we created 3D vibratome models of MOC2 and MOC2^CX3CL1^ tumors and immunostained them using double-fluorescence staining of CX3CL1 and PDPN. The structures of PDPN^+^ LVs were transformed entirely as CX3CL1 expression around the structures increased in MOC2^CX3CL1^ tumors, from the large tubular structures in MOC2 tumors to the network of smaller aberrant LVs in MOC2^CX3CL1^ tumors (Figure 4E and Supplemental Videos 1 and 2).

To identify the association between increased CX3CR1^+^ cells and PDPN^+^ structures, we analyzed the colocalization of CX3CR1^+^ and PDPN^+^ structures. CX3CL1 expression can be induced in inflamed lymphatic endothelial cells (LECs), attracting CX3CR1^+^ cells near CX3CL1^+^ LVs (25, 35). CX3CR1^+^ cells closely associated with PDPN^+^ LVs were also significantly increased in MOC2^CX3CL1^ tumors (26%) compared with that in MOC2 tumors (17%) (Figure 4, F and G).

Considering these findings, CX3CL1 can increase the formation of lymphatic structures, change LV integrity, and simultaneously promote CX3CR1^+^ recruitment through LVs, thus resulting in increased metastasis of cancer cells to cervical LNs via the lymphatic pathway.

### Cell migration, LN metastasis, and recruitment of CX3CR1 are canceled without the signal peptide and chemokine domains of CX3CL1.

**Figure 5:**
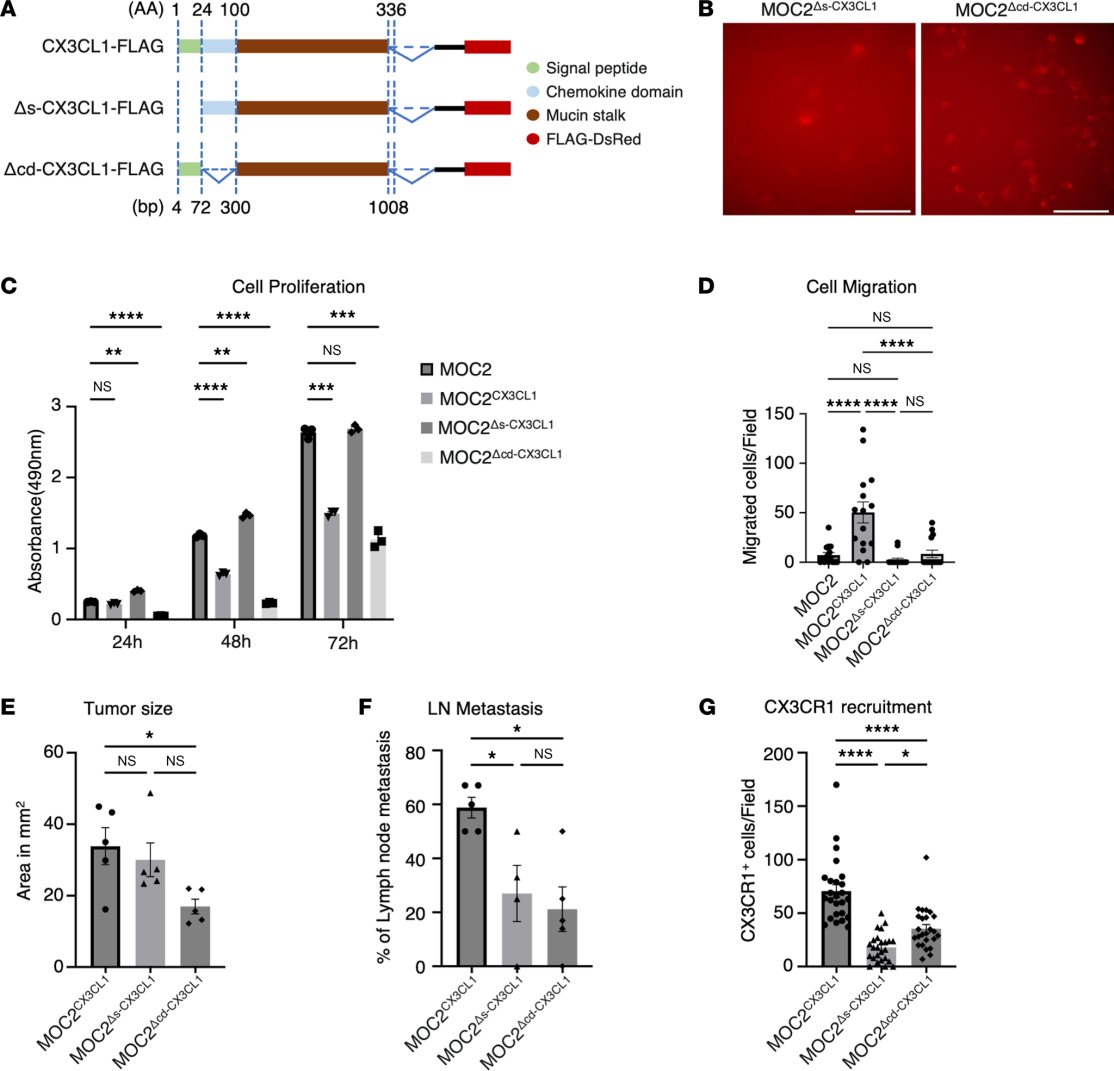
Cell migration, LN metastasis, and recruitment of CX3CR1 are eliminated without the signal peptide and chemokine domains of CX3CL1. The plasmids of signal peptide domain–deleted and chemokine domain–deleted CX3CL1 were constructed, and the plasmids were transfected to MOC cells. FLAG-DsRed was tagged to the plasmid for detection. (**A**) Schematic images showing the different isoforms of CX3CL1 tagged with FLAG-DsRed, soluble CX3CL1 overexpression (CX3CL1-FLAG), signal peptide–deleted CX3CL1 overexpression (Δs-CX3CL1-FLAG), and chemokine domain–deleted CX3CL1 overexpression (Δcd-CX3CL1-FLAG). Green: signal peptide, blue: chemokine domain, brown: mucin stalk, red: FLAG-DsRed. AA, amino acids; bp, base pairs. (**B**) Representative image of FLAG expression in MOC2^Δs-CX3CL1^ and MOC2^Δcd-CX3CL1^ cells. Scale bar: 100 μm. We checked the cell proliferation and migration rates without the signal peptide and chemokine domains in MOC cells. (**C**) Cell proliferation rate between 24 hours, 48 hours, and 72 hours. (*n* = 3.) (**D**) The number of migrating cells per field in MOC2, MOC2^CX3CL1^, MOC2^Δs-CX3CL1^, and MOC2^Δcd-CX3CL1^ cells. (*n* = 3.) (**E**) Tumor sizes measured by area in square millimeters. (**F**) The metastasis rate of cervical LNs. (**G**) The number of CX3CR1^+^ cells around the VLSs per field in MOC2^CX3CL1^, MOC2^Δs-CX3CL1^, and MOC2^Δcd-CX3CL1^ tumors. MOC2, MOC2 only; MOC2^CX3CL1^, MOC2 with CX3CL1 overexpression; MOC2^Δs-CX3CL1^, MOC2 with signal peptide–deleted CX3CL1; MOC2^Δcd-CX3CL1^, MOC2 with chemokine domain–deleted CX3CL1. MOC2^CX3CL1^: *n* = 5, MOC2^Δs-CX3CL1^: *n* = 5, MOC2^Δcd-CX3CL1^: *n* = 5. All data are presented as mean ± SEM. Statistical analysis was done using 1-way ANOVA followed by Tukey’s multiple-comparison post hoc test. *P < 0.05, **P < 0.01, ***P < 0.001, ****P < 0.0001.

The signal peptide and chemokine domain fragments of CX3CL1 have distinct functions (11). To further evaluate the involvement of the signal peptide domain and chemokine domain in OSCC metastasis, we constructed 2 additional plasmids, the signal peptide domain–deleted and the chemokine domain–deleted CX3CL1 plasmids, both of which were tagged with FLAG-DsRed for detection of expression (Figure 5A). The plasmids were transfected into MOC2 cells in the same manner as for MOC2^CX3CL1^ models. The transfected cells were selected using puromycin selection methods, and FLAG expression was checked under a confocal microscope to confirm the transfection effectiveness (Figure 5B).

To investigate the change in the effect of CX3CL1 on cancer cells without a signal peptide and chemokine domain in vitro, we performed MTS assay and Transwell migration assay on MOC2 cells transfected with signal peptide–deleted CX3CL1 plasmids (MOC2^Δs-CX3CL1^) and MOC2 cells transfected with chemokine domain–deleted CX3CL1 plasmids (MOC2^Δcd-CX3CL1^) and compared with the MOC2 and MOC2^CX3CL1^ groups. We found that inhibition of the cell proliferation effect in MOC2^CX3CL1^ was canceled out in MOC2^Δs-CX3CL1^ cells, while a decrease in cell proliferation could still be seen in MOC2^Δcd-CX3CL1^ cells (Figure 5C). The migration ability of the MOC2^Δs-CX3CL1^ and MOC2^Δcd-CX3CL1^ cells was reduced significantly compared with the MOC2^CX3CL1^ cells, returning to the state prior to CX3CL1 overexpression in MOC2 cells (Figure 5D).

These findings show that the signal peptide domain is a necessary component of CX3CL1 for decreasing cell proliferation and increasing cell migration, whereas the chemokine domain mainly affects the migratory ability of cancer cells.

To further investigate the in vivo effects of CX3CL1 without functional domains on the TME, we created orthotopic murine models again, in the same manner, using CX3CL1 domain–deleted MOC2 cells. We analyzed the changes in tumor growth and metastatic ability. MOC2^Δcd-CX3CL1^ tumors showed a significant reduction in tumor sizes compared with MOC2^CX3CL1^ tumors (Figure 5E). Cervical LN metastases of MOC2^Δs-CX3CL1^ and MOC2^Δcd-CX3CL1^ LNs were significantly reduced in rate compared with MOC2^CX3CL1^ LNs, which correlated with in vitro results (Figure 5F).

These results indicate that the signal peptide and chemokine domains are the necessary functional domains for the migration and metastasis of MOC2 cancer cells in vitro and in vivo.

To investigate whether the deletion of the functional domains of CX3CL1 affects the recruitment of CX3CR1^+^ cells into the TME, we evaluated the number of infiltrating CX3CR1^+^ cells in the TME. The functional domain–deleted models showed a significant reduction in the recruitment of CX3CR1^+^ cells into the TME compared with MOC2^CX3CL1^ tumor models (Figure 5G).

These data indicate that the functional domains of the CX3CL1 are also crucial for the recruitment of CX3CR1^+^ cells into the TME.

### The signal peptide and chemokine domains of CX3CL1 are the essential components for tumor lymphangiogenesis of aggressive, metastatic cancer.

**Figure 6:**
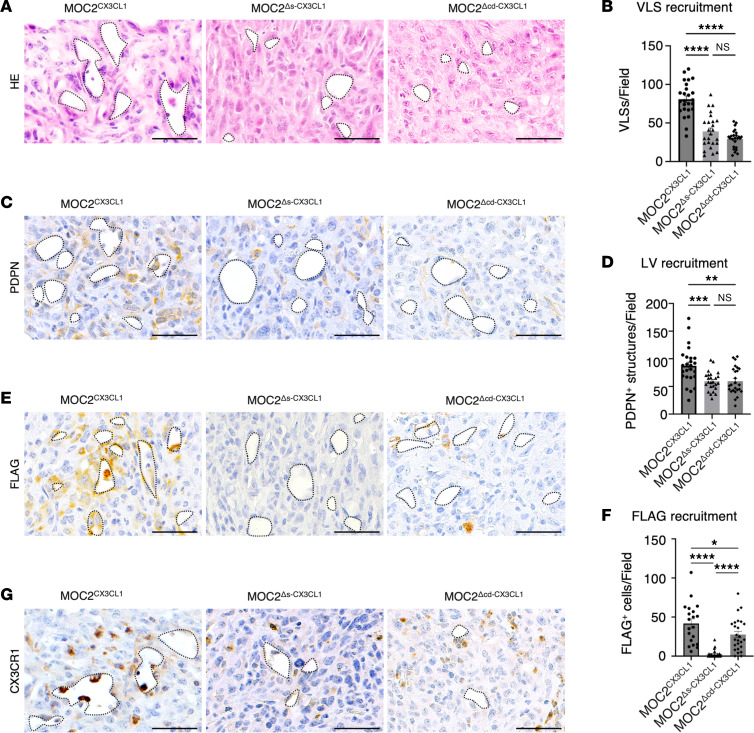
The signal peptide and chemokine domains of CX3CL1 are the essential components for tumor lymphangiogenesis of aggressive metastatic cancer. (**A**) Representative images and (**B**) the number of VLSs per field, (**C**) representative images and (**D**) the number of PDPN^+^ structures per field, (**E**) representative images and (**F**) the number of FLAG ^+^ cells around the VLSs per field, and (**G**) representative images of CX3CR1^+^ cells around the VLSs per field, in MOC2^CX3CL1^, MOC2^Δs-CX3CL1^, and MOC2^Δcd-CX3CL1^ tumors. (**A**, **C**, **E**, and **G**) Dotted lines represent the VLSs. Scale bar: 50 μm. MOC2^CX3CL1^: *n* = 5, MOC2^Δs-CX3CL1^: *n* = 5, MOC2^Δcd-CX3CL1^: *n* = 5. All data are presented as mean ± SEM. Statistical analysis was done using 1-way ANOVA followed by Tukey’s multiple-comparison post hoc test. *P < 0.05, **P < 0.01, ***P < 0.001, ****P < 0.0001.

To evaluate an association between the functional domains of CX3CL1 in cancer and the cervical LN metastasis and lymphangiogenesis, we analyzed the change in lymphatic vasculature landscape in MOC2^Δs-CX3CL1^ and MOC2^Δcd-CX3CL1^ tumors. First, we investigated whether the reduction in cervical LN metastases in MOC2^Δs-CX3CL1^ and MOC2^Δcd-CX3CL1^ was caused by the lymphatic pathway by counting VLSs in MOC2^Δs-CX3CL1^ and MOC2^Δcd-CX3CL1^ tumors. MOC2^Δs-CX3CL1^ and MOC2^Δcd-CX3CL1^ tumors significantly reduced VLSs compared with MOC2^CX3CL1^ tumors (Figure 6, A and B). Subsequently, we identified the population of lymphatic structures from VLS structures in MOC2^∆s-CX3CL1^ and MOC2^∆cd-CX3CL1^ tumors using PDPN marker. MOC2^Δs-CX3CL1^ and MOC2^Δcd-CX3CL1^ tumors had decreased PDPN^+^ VLSs compared with MOC2^CX3CL1^ tumors (Figure 6, C and D).

These results indicate that the lymphangiogenesis effect of CX3CL1 is deleted without functional domains of CX3CL1.

Next, to verify the correlation between LV structures, domains of CX3CL1, and CX3CR1, we analyzed the accumulated FLAG^+^ and CX3CR1^+^ structures around LVs. We found a significant decrease in FLAG^+^ structures around LV structures in the MOC2^Δs-CX3CL1^ and MOC2^Δcd-CX3CL1^ tumors compared with MOC2^CX3CL1^ tumors (Figure 6, E and F). Since the reduction of the CX3CR1^+^ structures in functional domain–deleted models was verified, we analyzed the histological location of the CX3CR1^+^ cells around the LVs. We found the accumulation of CX3CR1^+^ cells in MOC2^CX3CL1^ tumors was completely dissipated in MOC2^Δs-CX3CL1^ and MOC2^Δcd-CX3CL1^ tumors (Figure 6G).

These findings indicate that the functional domains of CX3CL1 are essential for the recruitment of CX3CR1^+^ structures and lymphangiogenesis of the TME and, thus, are responsible for cancer metastasis to cervical LNs via the lymphatics.

### CX3CL1 enrichment demonstrates a close correlation with lymphangiogenesis and poor prognosis of OSCC.

**Figure 7:**
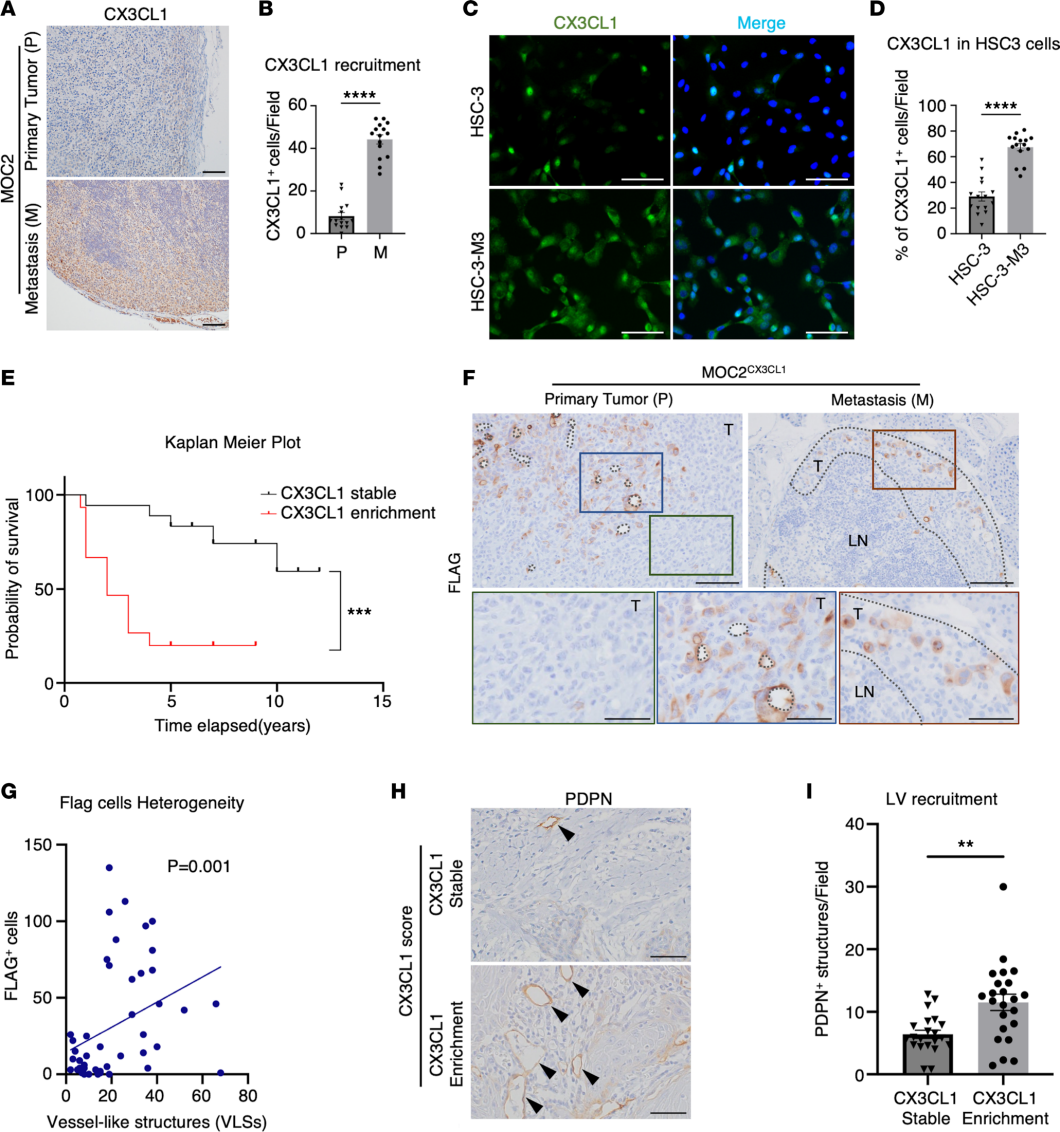
Expression “switching on” in the cervical LN metastasis tissues is closely associated with the overall survival in patients with OSCC. (**A**) Representative images and (**B**) the number of CX3CL1^+^ cells in MOC2 primary tumor (P) and metastasis (M) areas. Scale bar: 200 μm. (**C**) Representative images of fluorescence staining of CX3CL1 (green) in HSC-3 and HSC-3-M3 cells. Scale bar: 50 μm. (**D**) The rate of HSC-3 and HSC-3-M3 cells expressing CX3CL1 per field. (**E**) Kaplan-Meier survival curve of patients with OSCC compared between CX3CL1 stable and enrichment groups of patients with OSCC. (**F**) Representative images of FLAG^+^ expression accumulating around the VLSs in MOC2^CX3CL1^ tumors (blue inset, dotted line represents VLSs) while the surrounding tumor tissues (green inset) show no FLAG expression. FLAG^+^ structures are also seen in the secondary metastasis area in the LN (brown inset, dotted lines enclose the metastasized area). Scale bar: 100 μm, scale bar (insets): 50 μm. (**G**) Spearman’s correlation graph of FLAG^+^ cells’ accumulation around the VLSs in MOC2^CX3CL1^ tumors. Spearman’s *r* coefficient = 0.464. (**H**) Representative images and (**I**) the number of PDPN^+^ structures in CX3CL1 stable and enrichment groups of patients with OSCC. Arrows represent PDPN^+^ LVs. Scale bar: 100 μm. (*n* = 45.) MOC2^CX3CL1^: *n* = 5, MOC2^Δs-CX3CL1^: *n* = 5, MOC2^Δcd-CX3CL1^: *n* = 5. All data are presented as mean ± SEM. Statistical analysis was done using Student’s *t* test for comparison of 2 groups and log-rank test for analysis of the overall survival rate. **P < 0.01, ***P < 0.001, ****P < 0.0001.

The highly aggressive cancer, MOC2, exhibits higher CX3CL1 expression than the indolent cancer. Remarkably, within MOC2 tumors and lymphatic metastatic tissues, CX3CL1 expression in MOC2 tumors significantly increased in lymphatic metastatic sites (Figure 7, A and B). This enrichment of CX3CL1 at metastatic sites was further validated using the human OSCC cell lines HSC-3 and HSC-3-M3. HSC-3-M3, a highly metastatic OSCC cell line derived from HSC-3 cells (36), showed higher CX3CL1 expression than HSC-3 (Figure 7, C and D, and Supplemental Figure 5A). These findings suggest that CX3CL1 expression in OSCC increases during metastatic progression in both murine and human OSCC models.

To determine whether CX3CL1 could function as a potential marker of metastasis and poor prognosis in patients with aggressive OSCC, 45 patients with cervical LN metastasis underwent CX3CL1 immunostaining analysis of the primary tumor and metastatic LNs. CX3CL1 expression levels were scored (Supplemental Figure 5B). Although we expected the expression of CX3CL1 in metastatic LN (M) to be higher than the primary tumor (P), some cases showed similar expression or lower expression in metastatic LN compared to the primary tumor (M ≤ P). We noticed that CX3CL1 enrichment cases (M > P) exhibited a strong correlation with poor prognosis compared with stable cases (M ≤ P) (Figure 7E). Furthermore, the CX3CL1 enrichment group showed higher rates of recurrence and distant metastasis (Table 2). Interestingly, the difference in expression between primary tumors and LNs did not correlate with prognosis, emphasizing the significance of CX3CL1 enrichment (Supplemental Figure 5, C and D). These results indicated that CX3CL1 enrichment in metastatic LNs can serve as a potential marker of poor prognosis in OSCC.

Using the FLAG expression staining in MOC2^CX3CL1^ tissues, we found cancer heterogeneity in MOC2^CX3CL1^ tumors, where FLAG^+^ cells accumulated around VLSs (Figure 7F, blue insets), and some tumor areas lacked FLAG expression (Figure 7F, green insets). FLAG^+^ cells were also present in the metastasis area of cervical LNs (Figure 7F, brown insets). FLAG^+^ cells and VLSs showed a significantly positive association, indicating that a high population of FLAG^+^ cells was found around VLSs in MOC2^CX3CL1^ tumors (*P* = 0.001, Spearman’s *r* coefficient = 0.464) (Figure 7G).

These findings suggest that CX3CL1 expression within the TME is heterogeneous and is induced around the VLSs through the migration of a population of CX3CL1^+^ cancer cells to cervical LNs for metastasis.

To evaluate the association between increased CX3CL1 expression in cervical LN metastatic areas and tumor lymphangiogenesis in patients with OSCC, we analyzed and counted PDPN^+^ structures around OSCC cancer nests in primary tumors representing the invasive front of OSCC. Around the cancer nest regions, the CX3CL1 enrichment patient group exhibited an increased number of PDPN^+^ structures compared with CX3CL1-stable patients (Figure 7, H and I).

These results demonstrated that when lymphangiogenesis occurs around the tumor-invasive front, CX3CL1^+^ cells migrate through the LVs, enriching CX3CL1 expression in the metastatic LN area.

## Discussion

Recent studies showed that malignant tumors comprise a heterogenic population of cells where some cancers contain genotypes of good and bad prognoses in the same tumor (36). Our study used murine OSCC models with different tumor characteristics, aggressiveness, and immunophenotypes. MOC1 cells have desmosomal attachment structures, indicating a higher cell-to-cell attachment and less invasive properties in the cancer cells (24). Although they originated from the same origin, MOC2 cells lacked desmosomal attachment and showed higher invasive properties with metastasis to the cervical LNs. In addition, previous studies have shown that MOC1 cancer cells create an immune-inflamed TME, whereas MOC2 tumors are immune-excluded tumors with higher pro-tumor immune cell infiltration (20). However, chemokine responses to the TME are controversial because of their diverse roles in regulating immune cell transport and cancer cell activity. CX3CL1 was the focus of our study because of its multifaceted roles in cancer and the limited number of studies on CX3CL1 in OSCC (37–40).

Syngeneic murine models of different natures allowed us to understand the role of CX3CL1 in indolent and metastatic OSCC and the vast differences in their response to CX3CL1 chemokines. Interestingly, CX3CL1 overexpression produced similar results between indolent and aggressive cancer cells in the in vitro experiments. This outcome may be due to MOC1 and MOC2 cells originating from the same background (21). However, upon inoculation into mice, we discovered contrasting changes in tumor architecture between MOC1 and MOC2 tumors. CX3CL1 increase in MOC1 tumors resulted in smaller tumor size with increased tumor keratinization. In contrast, MOC2 tumors had higher infiltration of LVs and increased cervical LN metastasis. These findings lead us to consider the effects of CX3CL1 on the immune cells in the TME. In our study, we found that CX3CL1 expression can induce CX3CR1^+^ cells. CX3CR1 expression is positive in immune cells such as T cells, macrophages, and NK cells (41). We discovered that the antitumor immune system was promoted in MOC1^CX3CL1^ tumors, as demonstrated by recruitment of a higher number of cytotoxic T cells, alongside simulation of the pro-tumor immune system in MOC2^CX3CL1^ tumors when CD4^+^ T cells and Treg cells are recruited. The immune cell population recruited into the tumor differs because of the differences in immunophenotypes of MOC1 and MOC2 tumors, leading to the contrasting differences in the direction of how tissue architecture changes, and the variation in recruited immune population gave us an insight into how immune-inflamed and immune-excluded TME may react uniquely to the same chemokine (20, 42). Our findings showed the importance of considering the immune landscapes of cancer patients when selecting chemokine therapies.

LN metastasis is an indicator of poor prognosis in head and neck cancer (40). Cancer cells break away from the primary tumor and migrate to secondary metastasis sites via the blood or lymphatic system, forming new tumors in other body parts (43). The most common site for head and neck cancer metastasis is cervical LNs (44). The 5-year survival rate of patients with oral cancer declines from 75% to 49% when LN metastasis occurs (45). When the CX3CL1 is overexpressed in aggressive MOC2 tumors, the LN metastasis rate exponentially increases from 37% to 67%. This mechanism shows that CX3CL1 induces cervical lymphatic metastases. However, the underlying mechanism is still unclear.

Lymphangiogenesis in the primary tumors is an essential process in metastasis to LNs (46, 47). Our study revealed that when aggressive cancer cells gain high CX3CL1 expression, they can alter the vessel architecture of the primary tumor. We observed increased lymphatic structures in MOC2^CX3CL1^ tumors. This result suggests that CX3CL1 promotes cancer metastasis via the LVs. A detailed examination revealed that the lymphatic systems in MOC2^CX3CL1^ tumors were smaller and immature, branching out into complex structures and creating an aberrant lymphatic network. This suggests that cancer cells with high CX3CL1 expression (CX3CL1^hi^) could induce immature tumor lymphangiogenesis in the TME. In addition, CX3CL1 expression was induced in the LECs. CX3CL1 expression in the LECs may be induced directly or indirectly through DCs (35). CX3CL1 can promote the expression of CX3CR1 in cells through an autocrine process (25). Therefore, LECs expressing CX3CL1 might help induce the transendothelial migration of CX3CR1^+^ cells into LVs. We observed increased colocalization of CX3CR1^+^ cells and PDPN^+^ structures with increasing lymph-circulating tumor cells after CX3CL1 overexpression. This finding suggests that the transendothelial migration of the CX3CR1^hi^ cancer cells into the CX3CL1^+^ LVs ultimately promotes LN metastases.

Chemokines contain different molecular components that play essential roles in performing cell adhesion, migration, and leukocyte chemotaxis (48). N-terminal signal peptides perform signaling functions, leading the chemokine toward its receptors (49). In our data, without the signal peptide and chemokine domains in CX3CL1, migration effects in vitro and LN metastasis in vivo were inhibited, and the metastasis-aiding phenomena, such as lymphangiogenesis and recruitment of CX3CL1^+^ cells around lymphatics, diminished. This result implies that the signal peptide and chemokine domains are essential parts of CX3CL1 in metastasis. We also observed that tumor sizes become significantly smaller when chemokine domain was removed from CX3CL1 (Figure 5E). Moreover, there were differences in the number of recruited CX3CR1^+^ cells in the TME when the CX3CL1 ligand was expressed without signal peptide or chemokine domains. Further studies are required to identify the underlying phenomena. This study will be our team’s focus in the future.

Cervical LN metastasis can lower the overall survival rate of patients with OSCC and is treated with surgical removal of cervical LNs (45). However, the prognostic outcomes vary greatly, even within the patient population with lymphatic metastasis. In MOC2 tumors and their metastasis sites, we found that CX3CL1 expression was higher in the metastatic sites. Similarly, in human metastasis cancer cell lines, metastatic cell line HSC-3-M3, derived from HSC-3, showed increased CX3CL1 expression compared with HSC-3 cells. This indicated that CX3CL1 expression was intensified during metastasis progress in OSCC. We identified this phenomenon as “CX3CL1 enrichment.” In addition, CX3CL1 enrichment in patients with OSCC showed a strong correlation with a lower overall 12-year survival rate and higher distance of metastasis and recurrence rates. CX3CL1 expression enrichment in metastasis models indicates that the cancer character changed to a more aggressive nature. Thus, CX3CL1 enrichment in the metastasis LNs could be used as a clinical marker in patients with LN metastasis for long-term follow-up. In contrast, the expression differences in the primary tumor and LNs showed no correlation with the prognosis of patients with OSCC. Determining the outcomes of OSCC on the expression analysis alone can be tricky since cancer comprises a heterogeneous population of cells with multiple cancer subclones (36, 50). Each subclone can respond differently to chemokine therapy. This phenomenon has been demonstrated by CX3CL1 overexpression in indolent and aggressive murine cancer models, where MOC1 and MOC2 cancer subclones had different responses to CX3CL1 overexpression. Therefore, the difference in CX3CL1 expression might not represent the severity of the disease since it is challenging to determine which subclones of cancer gain CX3CL1 expression.

Chemokines and their receptors are expressed in endothelial cells and are involved in the recruitment and migration of cells through cell-to-cell contact or secretory signals (51). CX3CL1 overexpression tagged by FLAG^+^ cells was also found to be dispersed in a heterogenic manner and highly associated with the VLSs. These results suggested that CX3CL1^hi^ cells tended to accumulate around the VLSs for migration to cervical LNs. Interestingly, when CX3CL1 enrichment was seen in patients with OSCC, higher lymphatic structures were found near the invasive front of the primary tumor in OSCC samples. These findings suggest that CX3CL1 enrichment can serve as an indicator of tumor-induced lymphangiogenesis.

To the best of our knowledge, this is the first report on the influence of CX3CL1 on cervical LN metastasis in OSCC. CX3CL1 expression enrichment at the metastatic site can potentially be used as a prognostic predictor in OSCC with LN metastasis and can be used in long-term monitoring. Our research provides a better understanding of the dual roles of chemokines and their relationship with different cancer phenotypes, which can be used in a strategic approach to treating cancer in the near future.

## Methods

### Sex as a biological variable.

Our study examined both men and women with OSCC and accounted for this covariate in our analyses. There were no sex-specific differences in CX3CL1 expression in the patients. For this reason, sex is not considered as biological variable for our study. We restricted our mouse models to female for uniformity and cost purposes.

### Gene expression analysis.

The microarray data (GEO accession no. GSE50041) of MOC1 and MOC2 were available for analysis on the NCBI data bank. The DEGs were selected and analyzed using GEO2R software (https://www.ncbi.nlm.nih.gov/geo/geo2r/) and DAVID version 6.8. The Bioconductor limma software was used to investigate the expression level.

### Cell lines and mice.

MOC cell lines (MOC1; KER-EWL001-FP, MOC2; KER-EWL002-FP) were purchased from a Kerafast cell bank. These cells were cultured in Iscove’s modified Dulbecco’s medium (IMDM) (Gibco) and Ham’s Nutrient Mixture (F12) (Gibco) in a 2:1 ratio with 5% FBS (Gibco), 1% antimycotic-antibiotic solution (Anti-Anti) (Gibco), 5 mg/mL insulin (MilliporeSigma), 400 ng/mL hydrocortisone (MilliporeSigma), and 5 ng/mL EGF (585508 BioLegend) at 37°C in a humidifying incubator with 5% CO_2_. Human OSCC cell lines (HSC-3; JCRB0623, HSC-3-M3; JCRB1354) were purchased from the Cell Bank of the Japanese Collection of Research Bioresources. The cells were cultured in α-MEM (Gibco) supplemented with 10% FBS and 1% Anti-Anti at 37°C in a humidified incubator with 5% CO_2_.

Wild-type C57BL/6J female mice (6–8 weeks old) were purchased from Charles River Laboratories. All mice were housed in a standard animal facility with a pathogen-free environment.

### Antibodies.

The primary antibodies used were CX3CL1 (14-7986-81/Polyclonal Thermo Fisher Scientific), CX3CR1 (13885-1-AP/Polyclonal Proteintech), PDPN (015-24111/PMab-1 Wako), CD4 (25229S/D7D2Z Cell Signaling Technology), CD8 (98941S/D4W2Z Cell Signaling Technology), FOXP3 (ab215206/EPR22102-37 Abcam), CD34 (ab81289/EP373Y Abcam), and DYKDDDDK (14793S/D6W5B Cell Signaling Technology) for MOC cells and C57BL/6J wild-type mouse tissue sections. CX3CL1 (ab25088/Polyclonal Abcam) and PDPN (M3619/D2-40 Dako) were used for HSC-3 cells and human OSCC samples. The secondary antibodies used were chicken anti-rabbit IgG Alexa Fluor 488 (A21441/Polyclonal Thermo Fisher Scientific) and donkey anti-rat IgG Alexa Fluor 594 (A21209/Polyclonal Thermo Fisher Scientific).

### Reverse transcription and reverse transcription quantitative PCR.

TRI Reagent (Cosmo Bio) was used to extract the total RNA. Extracted RNA concentration was measured using Life Science UV/Vis Spectrometer (DU 730), and the concentration was adjusted. RNA was reverse-transcribed by PrimeScript reverse RT Master Mix (Takara) to cDNA. A LightCycler 96 System (Roche) was used to determine the quantity of RNA. The default setting on the LightCycler96 software automatically selected the Ct. The sequences of the target genes were as follows: *Gapdh*-forward, 5′-AACTCCCACTCTTCCACCTTC-3′, *Gapdh*-reverse, 5′-CCTGTTGCTGTAGCCGTATTC-3′; and *Cx3cl1*-forward, 5′-ATCCCAGTGGCTTTGCTCAT-3′, *Cx3cl1*-reverse, 5′-ATTTCTCCTTCGGGTCAGCA-3′ for mouse OSCC cell analysis and *GAPDH*-forward, 5′-CTCATGACCACAGTCCATGC-3′, *GAPDH*-reverse, 5′-TTACTCCTTGGAGGCCATGT-3′; and *CX3CL1*-forward, 5′-GCCACATTCCTGATGCTTCT-3′, *CX3CL1*-reverse, 5′-AACTTCCCCTTTCCCATGTC-3′ for human OSCC cell analysis. The difference in gene expression levels between the cell lines was calculated using the 2^−ΔΔCt^ method.

### Plasmid design and gene transfection.

To subclone several expression genes, the primers listed in Supplemental Table 1 were used to amplify the genes by PCR, and subsequently, plasmids were successfully constructed using the Gibson Assembly method in this study. In brief, the DsRed fragment amplified by PCR with primer Fw1 and Rv1 using pDsRed-monomer vector (632467 Clontech, Takara) as a template was inserted into p3×FLAG-CMV13. Next, a coding region of the soluble form of murine *Cx3cl1* was amplified by PCR with primer Fw2 and Rv2 using mouse *Cx3cl1* cDNA (MGC clone ID: 3498747, MMM1013-202762071 Dharmacon), as a template, and a coding region of DsRed-3×FLAG was amplified by PCR with primer Fw3 and Rv3 using p3×FLAG-CMV13-DsRed as a template. The fragments, CX3CL1 and DsRed-3×FLAG, were subcloned into pLVSIN-EF1α Pur vector (6186 Takara), which was a lentivirus gene expression vector possessing CX3CL1 fused with DsRed-3×Flag at its C-terminus and was represented by pLEp-CX3CL1-Flag. Constructions of functional domain–deleted models of CX3CL1 were performed by PCR using pLEp-CX3CL1-Flag as the template with the PrimeSTAR Mutagenesis Basal Kit (R046A Takara) according to the manufacturer’s instructions. The primers used were Fw4 and Rv4 for Δs-CX3CL1 and Fw5 and Rv5 for Δcd-CX3CL1, respectively. MOC1 and MOC2 cells were seeded in 6-well plates (1.5 × 10^5^ cells/well). A stable cell line of CX3CL1-overexpressed MOC cells was established with the lentivirus gene expression system. The puromycin kill curve was performed using puromycin concentration from 0.5 μg/mL to 3 μg/mL. A stable selection of transfected cells was achieved with optimal puromycin concentration. The puromycin-resistant clones continued to culture in 1 μg/mL puromycin.

### Transwell migration assay.

MOC cells were resuspended in the IMDM without FBS at 1 × 10^5^ cells/300 μL concentration and placed inside the upper inserts with 8 μm pore size. IMDM with 10% FBS was added into the lower chamber. After 12–24 hours of incubation at 37°C with 5% CO_2_, Giemsa staining was performed using Diff-Quik solution (Sysmex), and nonmigrated cells from the upper inserts were removed with cotton swabs. The photographs of the migrating cells were taken using an upright microscope BX53 (Olympus). The migrated cells were counted using Fiji-2 (Version 1.0).

### MTS assay.

The cells were seeded into 96-well plates at 1,500 cells per well in 100 μL medium. Following the incubation period of 24 hours, 48 hours, and 72 hours at 37°C, 20 μL of Cell Titer 96 Aqueous One Solution Cell Proliferation Assay (Promega) was added to each well, and the cells were incubated for a further 3 hours at 37°C with 5% CO_2_. The absorbance of each well was measured at 490 nm using an ELISA reader (Corona SH-1000Lab).

### Orthotopic OSCC mouse models.

Tumor transplantation was done to the right buccal mucosa of wild-type mice at the concentration of 3 × 10^4^ cells/50 μL HBSS (Invitrogen, Thermo Fisher Scientific) for MOC2 and MOC2^CX3CL1^ and 2 × 10^6^ cells/60 μL 1:3 ratio of Matrigel (Corning) to HBSS for MOC1 and MOC1^CX3CL1^. The mice were sacrificed after 3 weeks for MOC2-transplanted mice and 4 weeks for MOC1-transplanted mice.

For vibratome-sectioned tissue, MOC2 and MOC2^CX3CL1^ cells were injected into the head of the C57BL/6J mice at the concentration of 1 × 10^6^/100 μL to avoid the decalcification step. The specimens were collected for analysis.

### Tissue processing for histological examination.

The head bearing the tumor, the draining cervical LNs, and the lungs were harvested and fixed in 4% paraformaldehyde solution for 36–48 hours. The bone tissues were decalcified in Osteosoft (MilliporeSigma) at room temperature (RT) for 10–14 days until the tissues were softened enough for cutting. The tumors were cut at the largest cross-sectional areas. The tissues underwent a dehydration process starting at 70% ethanol and gradually increasing up to 100% alcohol, and xylene was used as a clearing agent before embedding in the paraffin. The tissues were cut at a 3 μm–thick cross section and analyzed using H&E, IHC, and immunofluorescence staining.

### IHC staining.

The samples were blocked with 1.2% hydrogen peroxide/methanol solution to inhibit endogenous peroxidase activity. The antigen retrieval was performed according to the manufacturer’s instructions for antibodies. After the antigen retrieval, the tissue slides were blocked with protein block for 30 minutes at RT and incubated with the primary antibody at 4°C overnight. The slides were incubated in secondary antibodies for 30 minutes at RT, followed by incubation in avidin-biotin complex (Vector Laboratories) for 30 minutes at RT. The color development was achieved by applying a 3,3′-diaminobenzidine (DAB) solution. The DAB incubation time was always determined by the pilot slides. Mayer’s Hematoxylin (MilliporeSigma) was used as a counterstain, dehydrated with the increasing alcohol concentration, and then added to xylene. The staining results were observed with an optical microscope (BX53, Olympus).

### Double-fluorescent IHC staining.

After the antigen retrieval, the tissues were incubated using Block Ace (DS Pharma Biomedical) for 20 minutes at RT. The primary antibody incubation was done at 4°C overnight. TBS washing was done and the secondary antibody incubation was done for 1 hour. The sections were stained with nuclear stain DAPI (Dojindo Laboratories). The staining results were observed with All-in-One BZ x700 fluorescence microscope (Keyence).

### Immunocytochemistry staining.

Cells were seeded into the 6-well plates at the concentration of 3 × 10^5^ cells per well. After 24 hours, the cells were fixed with 4% paraformaldehyde for 10 minutes. The cells were washed in 0.5% Tween 20 solution for 5 minutes and 0.05 M TBS. The cells were blocked using Block Ace (DS Pharma Biomedical) for 20 minutes at RT and incubated with primary antibodies for 2 hours. TBS wash was performed 3 times followed by incubation with secondary antibodies for 1.5 hours. The cells were then washed with TBS and distilled water. The sections were stained with nuclear stain DAPI. The staining results were observed with All-in-One BZ x700 fluorescence microscope.

### Vibratome tissue sectioning, staining, and imaging.

Freshly cut tumor tissues were embedded in 2% low-melt agarose. The tissues were cut into 100 μm thickness using a vibratome (Neo Linear Slicer, Dosaka EM). The tumor tissues were fixed overnight in a 2% phosphate-buffered formaldehyde solution before sectioning and staining. The tissue underwent permeabilization by 2% Triton X-100 and 0.05% NaN_3_ in 2% PBS-Tween solution. The sections were blocked with blocking buffer (10% FBS, 1% Triton X-100, 0.2% NaN_3_) overnight and incubated in the primary antibodies mouse CX3CL1 and mouse PDPN previously stated in *Antibodies* at RT for 2 nights. The sections were incubated in the secondary antibodies at RT overnight. The secondary antibodies used were Green anti-rabbit IgG (Alexa Fluor 488, Thermo Fisher Scientific) and Red anti-rat IgG (Alexa Fluor 594, Thermo Fisher Scientific) previously stated in *Antibodies*. The sections were washed 3 times for 30 minutes in washing buffer (3% NaCl, 0.2% Triton X-100 in PBS) between each step. The sections were incubated in the DAPI solution for 1.5 hours at room temperature. Sections were then washed with PBS solution and cleared using RapiClear 1.47 solution for 60 minutes, and we mounted the slides with a clear fluorescence mounting medium (Dako). The staining results were observed using a confocal microscope (ZEISS LSM780).

### Interpretation of IHC staining of OSCC sample.

The selection criteria for the 45 patients with OSCC were OSCC with positive LN metastasis with no prior cancer treatment. The representative tumor and LN tissue sections with the secondary metastasis from each patient were selected. The staining results were observed with an optical microscope (BX53, Olympus). The stained areas were then analyzed and interpreted using a weighted histoscore by 2 pathologists under a protocol blinded to clinical outcomes to eliminate the potential bias. The expression levels of CX3CL1 in cancer cells were arranged into 5 groups, 0 being negative, 1 being 5% of the cells with positive staining, 2 being between 5% and 50% of the cells with positive staining, 3 being more than 50% of the cells with weak staining, and 4 being more than 50% of the cells with strong staining (52). The scores of 0 and 1 were defined as low-expression groups and the scores of 2, 3, and 4 as high-expression groups. Kaplan-Meier plot of 45 patients with OSCC was constructed using GraphPad Prism 9.1.1.

### Statistics.

All the data were presented as the mean ± SEM. All the in vitro experiments were independently repeated 3 times. Five manually placed original magnification, ×40, regions were used to analyze the tissue sections. The positive cells were counted using Fiji-2 (Version 1.0). Two-tailed Student’s *t* tests were used to compare 2 groups, and 1-way ANOVA with multiple-comparison tests were used among more than 3 groups. Kaplan-Meier survival curves of OSCC patient samples were compared using the log-rank test. The χ^2^ test was used to evaluate the relationship between CX3CL1 expression and clinicopathological characteristics. Spearman’s rank correlation coefficient was used to analyze the association between FLAG^+^ structures and VLSs. Statistical analyses were performed using GraphPad Prism 9.1.1. *P* values less than 0.05 were considered significant.

### Study approval.

All animal experiments were done under the guidelines of the Okayama University Care and Use of Laboratory Animals. The research was approved by the Committee on the Ethics of Animal Experiments of Okayama University Graduate School of Medicine, Dentistry and Pharmaceutical Sciences (OKU-2020096). Human tissues were obtained from patients at the Department of Oral Pathology and Medicine, Okayama University Graduate School of Medicine, Dentistry and Pharmaceutical Sciences, from 2010 to 2020. The Okayama University Ethics Committee approved the study (Clinical Ethics number-2008-032) and waived written informed consent, as patient samples were deidentified and patients were provided the opportunity to refuse participation.

### Data availability.

The GEO data analyzed in this study are available under the accession no. GSE50041. The raw values of MOC1 and MOC2 cells are available for download from https://www.ncbi.nlm.nih.gov/geo/query/acc.cgi?acc=GSE50041 All the data value points in graphs are reported in the Supporting Data Values file.

## Author contributions

HK conceptualized and designed the study. HK and HSE wrote the original draft of the manuscript. TO, MWO, and HN contributed to the manuscript writing. MN and HSE performed plasmid transfection. HK, HSE, YF, MWO, KO, and YS performed experiments. KT, QS, and HSE performed data analysis. HN, SI, NM, and KN contributed and oversaw the experimental designs. HK, KT, KN, and HN acquired funding. HN and SI supervised HK and HSE. All authors have read and agreed to the published version of the manuscript.

## Supplementary Material

Supplemental data

Supplemental video 1

Supplemental video 2

Supporting data values

## Figures and Tables

**Table 1:**
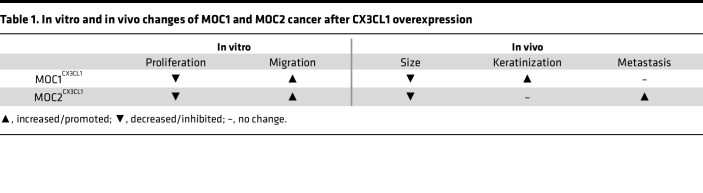
In vitro and in vivo changes of MOC1 and MOC2 cancer after CX3CL1 overexpression

**Table 2:**
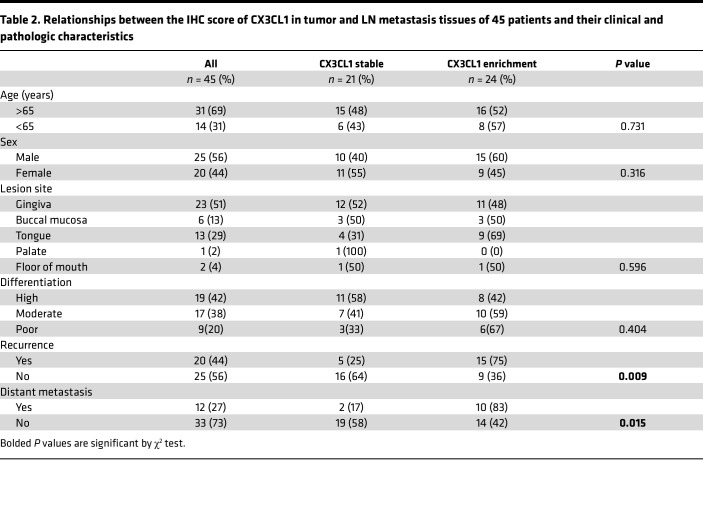
Relationships between the IHC score of CX3CL1 in tumor and LN metastasis tissues of 45 patients and their clinical and pathologic characteristics

## Version 1 05/22/2024

Electronic publication
