# Supplementary material for: Double-faced CX3CL1 enhances lymphangiogenesis-dependent metastasis in an aggressive subclone of oral squamous cell carcinoma
Source: JCI Insight. 2024 May 22;9(10):e174618. doi: 10.1172/jci.insight.174618 (PMC11141908; doi:10.1172/jci.insight.174618)
Supplement: Supplemental data [file jciinsight-9-174618-s039.pdf]

Supplementary Figure legends

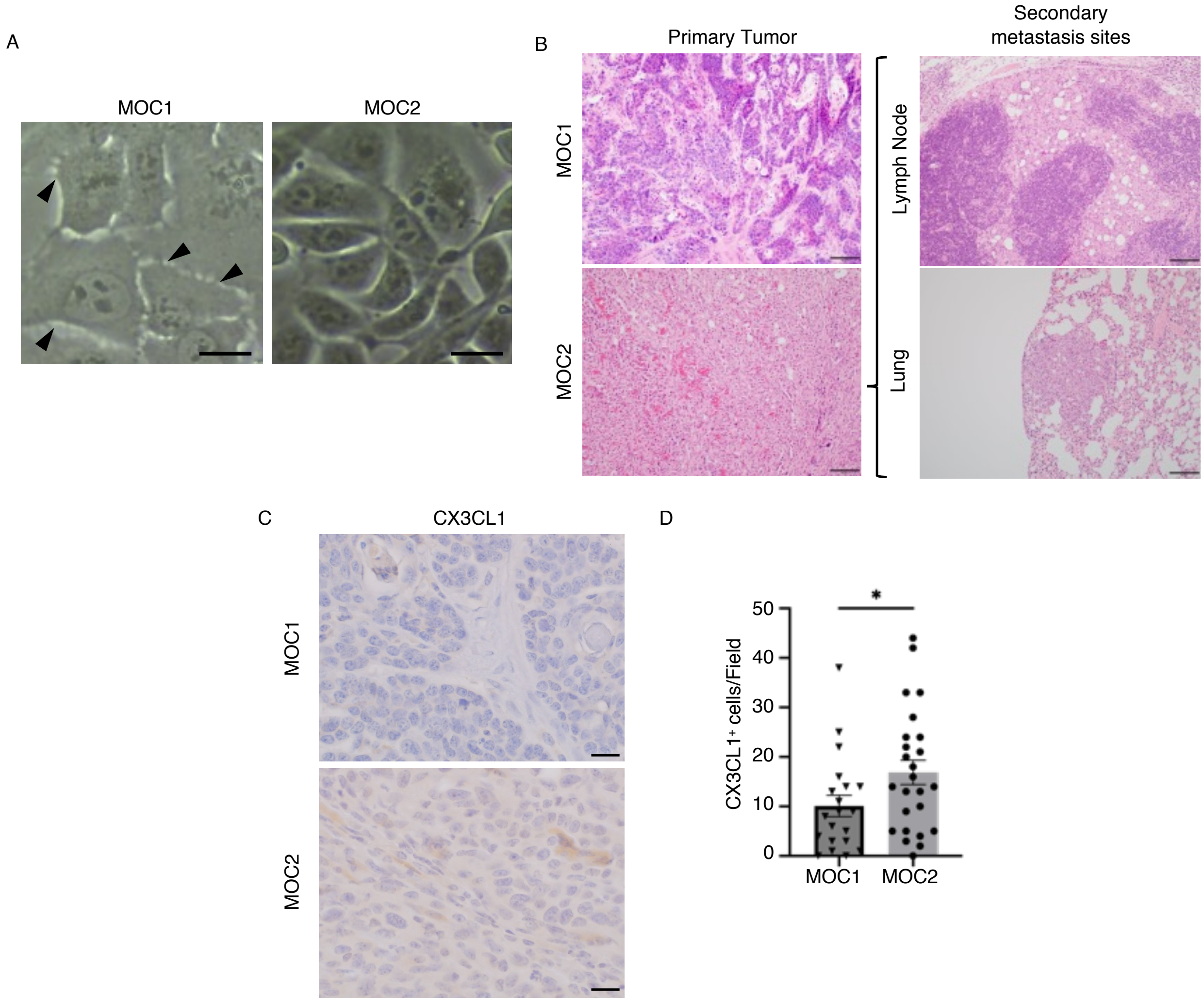

Supplementary Figure S1. Characteristics of MOC1 and MOC2, and higher CX3CL1 expression in primary tumors of MOC2.

(A) Representative images showing MOC1 and MOC2 cells. The arrows indicate the desmosomal attachment structures of MOC1 cells. Scale bar: 20µm. **(B)** Representative images showing primary tumors of MOC1 and MOC2 and the secondary metastasis tissues (lymph node and lungs) of MOC2. MOC1 is well-differentiated in nature and indolent cancer without metastasis ability. MOC2 is poorly differentiated and aggressive, with naturally occurring cervical lymph nodes and lung metastasis. Scale bar: 100µm. **(C)** Representative images and **(D)** the number of $CX3CL1^{+}$ cells per field in MOC1 and MOC2 tumors.
MOC1: n=4, MOC2: n=5.
All data are presented as mean±SEM. Statistical analysis was done using Student's t-test.
*$p<0.05$.

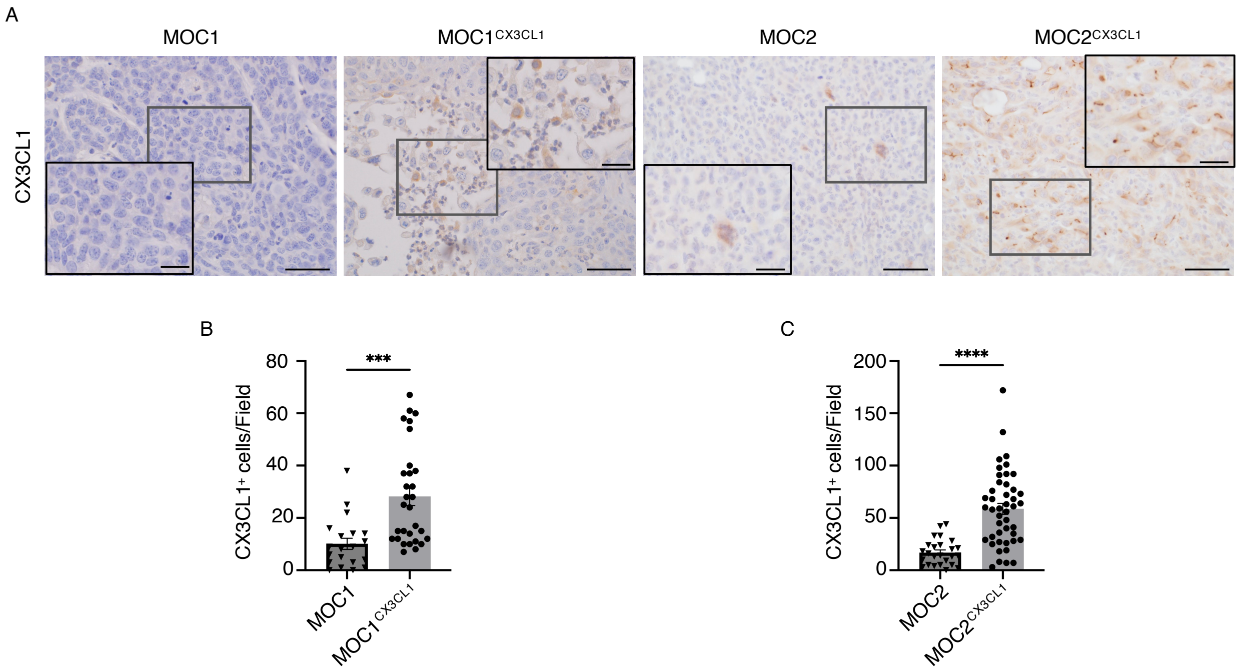

Supplementary Figure S2. CX3CL1⁺ cells after overexpression in vivo.
(A) Representative images showing $CX3CL1^{+}$ cells in MOC and MOC^{CX3CL1} primary tumors. Scale bar: 50µm. The magnified images of the $CX3CL1^{+}$ areas are shown in the insets. Scale bar (insets): 20µm. The number of $CX3CL1^{+}$ cells per field in **(B)** MOC1 and $MOC1^{CX3CL1}$ tumors and **(C)** MOC2 and $MOC2^{CX3CL1}$ tumors.
MOC1: n=4, $MOC1^{CX3CL1}$: n=6, MOC2: n=5, $MOC2^{CX3CL1}$: n=9.
All data are presented as mean±SEM. Statistical analysis was done using Student's t-test.
***p<0.001, ****p<0.0001.

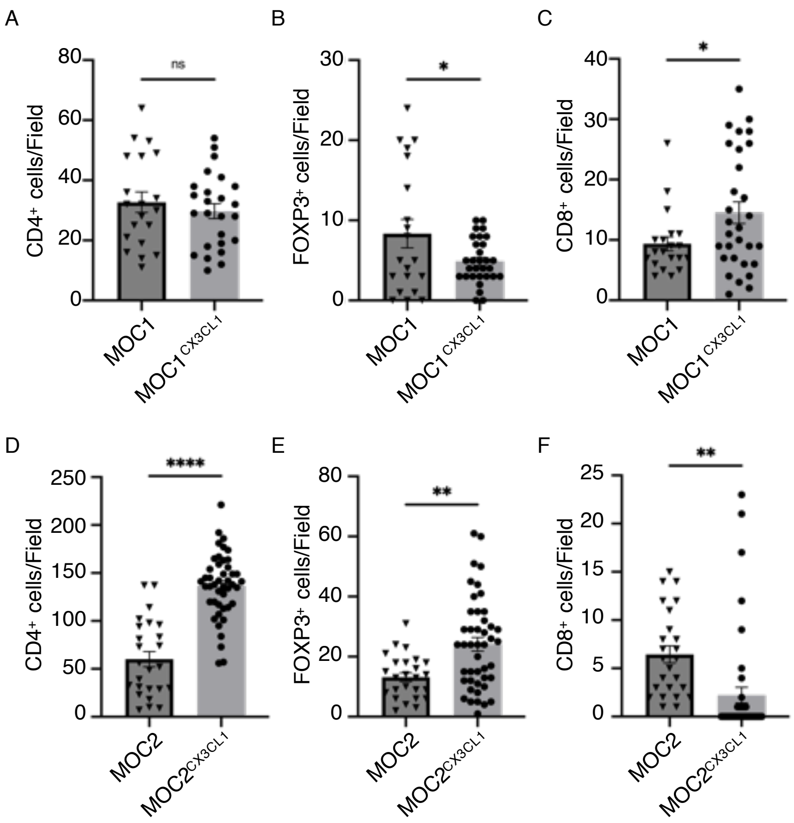

Supplementary Figure S3. Different populations of recruited T cells into the TME of MOC1 and MOC2 tumors after CX3CL1 overexpression.

Changes in the immune landscape of both MOC tumors were observed. The number of **(A, D)** $CD4^+$ cells, **(B, E)** $FOXP3^+$ cells, and **(C, F)** $CD8^+$ cells per field in **(A-C)** MOC1 and $MOC1^{CX3CL1}$ tumors and **(D-F)** MOC2 and $MOC2^{CX3CL1}$ tumors.

MOC1: n=4, $MOC1^{CX3CL1}$: n=6, MOC2: n=5, $MOC2^{CX3CL1}$: n=9.

All data are presented as mean±SEM. Statistical analysis was done using Student's t-test. ns: not significant, *p<0.05, **p<0.01, ****p<0.0001.

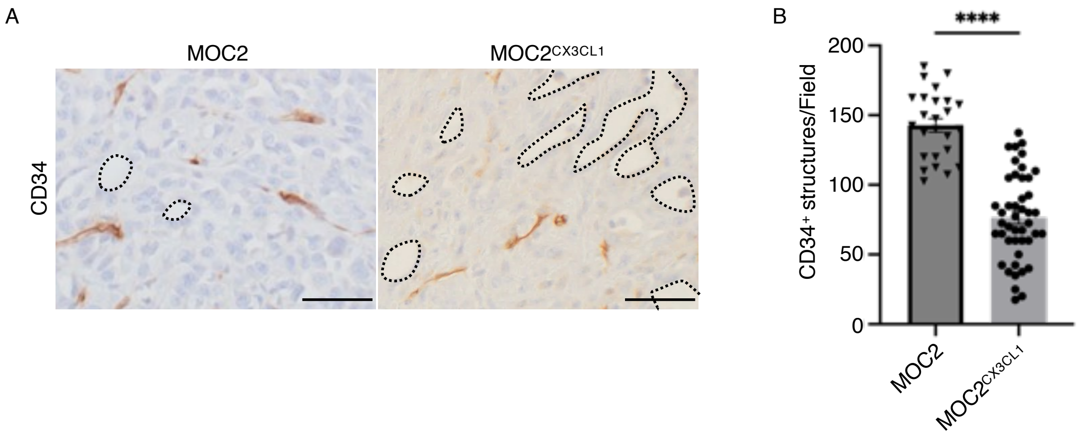

Supplementary Figure S4. CD34^{+} blood vessel structures have little to no association with increasing vessel-like structures in overexpression tumors.
(A) Representative images showing CD34^{+} structures around vessel-like structures in MOC2 and MOC2^{CX3CL1} tumors. Dotted lines represent the vessel-like structures. Scale bar: 50µm. **(B)** The number of CD34^{+} structures around the vessel-like structures per field in the MOC2 and MOC2^{CX3CL1} tumors.
MOC2: n=5, MOC2^{CX3CL1}: n=9.
All data are presented as mean±SEM. Statistical analysis was done using Student's t-test.
****p<0.0001.

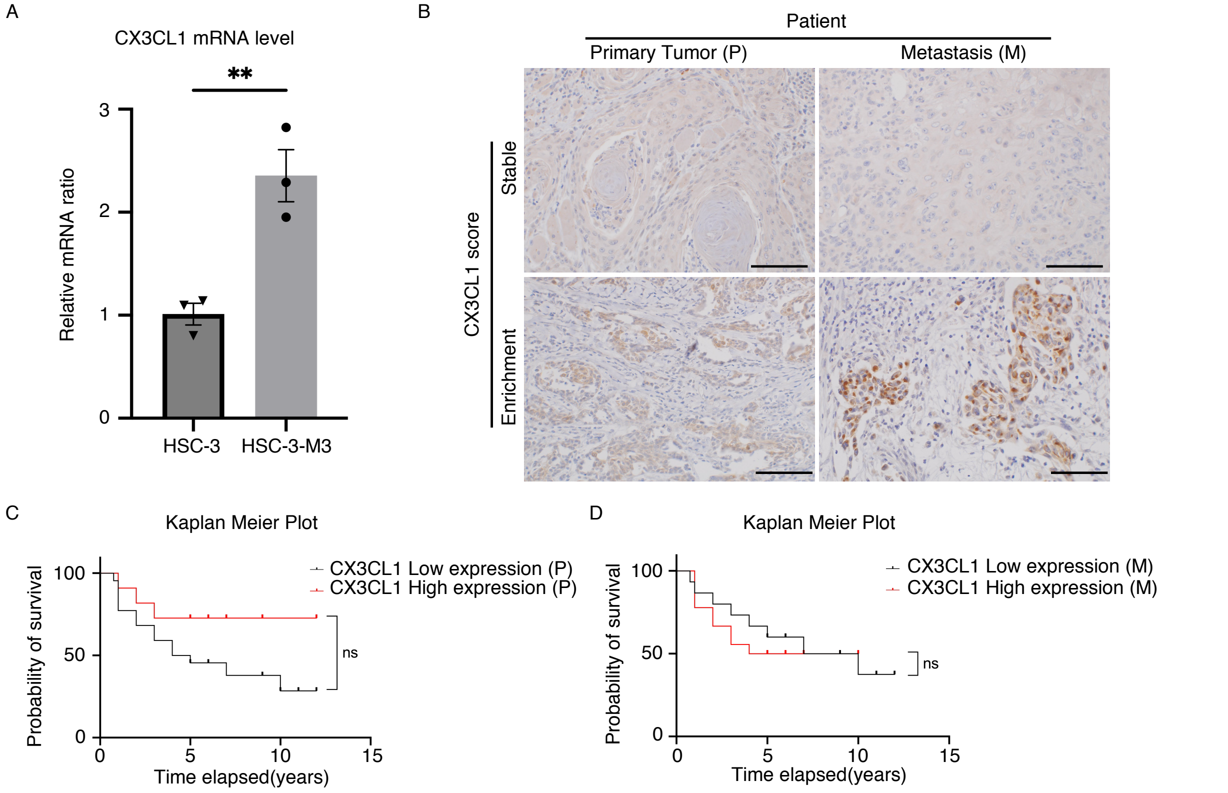

Supplementary Figure S5. CX3CL1 expression in human cell line and overall survival rate of OSCC patients in CX3CL1 expression high and low groups.

(A) Relative mRNA expression of *CX3CL1* in HSC-3 vs. HSC-3-M3 was determined by quantitative reverse-transcriptase PCR (RT-qPCR). *CX3CL1* expression was analyzed by the $2^{-\Delta\Delta Ct}$ method. *CX3CL1* expression level in HSC-3 was set as 1. (n=3) **(B)** Representative images showing CX3CL1 positive staining in primary (P) and metastasis (M) tumors of OSCC patients. CX3CL1 Stable: metastasis cancer cells have CX3CL1 expression scores lower or no change to the primary tumor cells. CX3CL1 Enrichment: metastasis cancer cells have CX3CL1 expression scores higher than the primary tumor cells. Scale bar:100μm. (n=45) **(C)** Kaplan-Meier survival curve of patients with OSCC compared between CX3CL1 expression of tumor tissues. **(D)** Kaplan-Meier survival curve of patients with OSCC compared between CX3CL1 expression of lymphatic metastatic tissues.

All data are presented as mean±SEM. Statistical analysis was done using Students' t-tests and log-rank tests for analysis of the overall survival rate. ns: **p<0.01, ****p<0.0001.

Primer	Target gene	Sequence (5'-3')
Fw1	DsRed	GTTTAGTGAACCGTCAGAATTATGGACAACACCGAGGACGTCATCAAGG
Rv1	DsRed	GTCATGGTCTTTGTAGTCAGCCCGGGATCCCTGGGAGCCGGAGTGGCGGGCCTCGGC
Fw2	CX3CL1	CGGAATTAGCTTGGTACGGGATCCATGGCTCCCTCGCCGCTCGCGTGGCTGC
Rv2	CX3CL1	GCGCGCGTCCTGCAGGACCTCGAGTGTGGCTGCCTGGGTGTCGGGGACAGG
Fw3	DsRed-3×Flag	CTCGAGGTCCTGCAGGACGCGCGCAATCGATGTCTAGAGATGGACAACACCGAGGACGTC
Rv3	DsRed-3×Flag	CAGGGATGCCACCCGGGATCACTACTTGTCATCGTCATCCTTGTAGTCGATG
Fw4	Δs-CX3CL1-Flag	CGGAATTAGCTTGGTACGGGATCCATGCAGCACCTCGGCATGACGAAATGC
Rv4	Δs-CX3CL1-Flag	GCATTTCGTCATGCCGAGGTGCTGCATGGATCCCGTACCAAGCTAATTCCG
Fw5	Δcd-CX3CL1-Flag	CCATTTGTGTACTCTGCTGCCGGGTGGCAAGTTTGAGAAGCGGGTGGAC
Rv5	Δcd-CX3CL1-Flag	GTCCACCCGCTTCTCAAACTTGCCACCCGGCAGCAGAGTACACAAATGG

Supplementary Table S1. List of Primer used for plasmid construction of CX3CL1 overexpressed MOC cell lines and CX3CL1 functional domain deleted MOC cell lines.
